# Cross-species transcriptomic atlas of dorsal root ganglia reveals species-specific programs for sensory function

**DOI:** 10.1038/s41467-023-36014-0

**Published:** 2023-01-23

**Authors:** Min Jung, Michelle Dourado, James Maksymetz, Amanda Jacobson, Benjamin I. Laufer, Miriam Baca, Oded Foreman, David H. Hackos, Lorena Riol-Blanco, Joshua S. Kaminker

**Affiliations:** 1 Department of OMNI Bioinformatics, Genentech, Inc., South San Francisco, CA USA; 2 Department of Neuroscience, Genentech, Inc., South San Francisco, CA USA; 3 Department of Immunology Discovery, Genentech, Inc., South San Francisco, CA USA; 4 Department of Pathology, Genentech, Inc., South San Francisco, CA USA

**Keywords:** Pain, Sensory processing

## Abstract

Sensory neurons of the dorsal root ganglion (DRG) are critical for maintaining tissue homeostasis by sensing and initiating responses to stimuli. While most preclinical studies of DRGs are conducted in rodents, much less is known about the mechanisms of sensory perception in primates. We generated a transcriptome atlas of mouse, guinea pig, cynomolgus monkey, and human DRGs by implementing a common laboratory workflow and multiple data-integration approaches to generate high-resolution cross-species mappings of sensory neuron subtypes. Using our atlas, we identified conserved core modules highlighting subtype-specific biological processes related to inflammatory response. We also identified divergent expression of key genes involved in DRG function, suggesting species-specific adaptations specifically in nociceptors that likely point to divergent function of nociceptors. Among these, we validated that *TAFA4*, a member of the druggable genome, was expressed in distinct populations of DRG neurons across species, highlighting species-specific programs that are critical for therapeutic development.

## Abstract (web summary)

Sensory neurons are critical for maintaining tissue homeostasis. Here, the authors generated a single-nuclei cross-species atlas of the dorsal root ganglia, revealing conserved programs for sensory function that could inform therapeutic hypotheses.

## Introduction

The dorsal root ganglion (DRG) plays a key role in conveying sensory information that leads to perception. A primary function of sensory neurons within the DRG is the detection of stimuli such as touch, noxious stimuli, temperature, itch, or proprioception, and to transmit this information to the central nervous system^1–3^. This ability to precisely differentiate and process distinct sensory information is enabled by the differential expression of ion channels, GPCRs, and other signaling receptors. Additionally, sensory neuron subtypes can differ in cell body size, degree of myelination, conduction velocities, and innervation tissue^4,5^.

Rodents have been the primary model system used to study somatosensory function. Unbiased descriptions of the rodent sensory neurons using single-cell RNA sequencing have provided opportunities to map transcriptional traits to anatomical and functional properties^6–8^. From these studies, rodent DRG neurons have been molecularly classified into different groups: heavily myelinated limb proprioceptors and A-fiber low-threshold mechanoreceptors (LTMRs) that express neurotrophin receptor tyrosine kinases (*Ntrk2* and *Ntrk3*); C-fiber LTMRs that express tyrosine hydroxylase (*Th*) and *Vglut3* (*Slc17a8*); C-fiber non-peptidergic nociceptors marked by the expression of *Mrgprd, Mrgpra3*, and *Sst*; non-myelinated C-fiber and lightly myelinated A$\delta$-fiber peptidergic nociceptors which express a variety of neuropeptides including substance P (*Tac1*), calcitonin related peptide (*Calca*) and pituitary adenylate-cyclase-activating neuropeptide (*Adcyap1*) along with *Ntrk1*; A$\delta$-fiber and C-fiber nociceptors that express the cooling and menthol sensing receptor, *Trpm8*. In rodent models, these markers correlate well with previous electrophysiological and neurochemical characterization and have enabled us to broaden our basic understanding of somatosensation.

However, even with our extensive understanding of rodent sensory neurons, the translation of somatosensory mechanisms from preclinical models to the clinic remains challenging due to molecular differences between sensory neurons present in rodents and humans. For example, a considerable subpopulation of neurons that co-express *NTRK1* and *RET* were observed in human DRGs, but were absent from mouse DRGs^9^. Additionally, numerous studies have highlighted that *SCN10A* (Na_v_1.8), *SCN11A* (Na_v_1.9), *P2X3* receptor, and *TRPV1* are essentially present in all human DRG neurons^10–12^, whereas in mice these genes are expressed in specific sub-populations of DRG neurons^8,13^. These differences between species accentuate the need for comprehensive, cross-species molecular studies of sensory neurons within the DRG.

Recent transcriptome studies have enabled a deeper understanding of the molecular landscape of primate DRGs^14–16^. However, differences in sequencing technologies, laboratory protocols, or sample archival methods between studies can make it challenging to interpret sensory neuron subtype mapping across species. These differences introduce technical artifacts that make it challenging to precisely integrate such transcriptome data from different species, which is crucial for the comparison of transcriptional programs across pre-clinical and clinical specimens. Various computational methods have been applied to align homologous cell types between species while attempting to address technical artifacts. These include Seurat for aggregating single-cell RNA-seq datasets using anchors identified from canonical correlation analysis^17^ and MetaNeighbor for quantifying cell-type replicability across data sets^18^. While studies have demonstrated the utility of these methods in cross-species cell-type homology mapping^19–21^, interpretation and evaluation of their performance require significant computational and biological expertise.

Ultimately, leveraging laboratory protocols and technologies with the most relevant computational methods is of utmost importance for the creation of meaningful, high-resolution cross-species mappings at a single-cell resolution. Mappings between preclinical models and humans are fundamental for characterizing gene expression similarities and differences between species to inform relevant therapeutic hypotheses.

Numerous studies have highlighted the therapeutic potential for targeting the neuro-immune axis^22–30^. For example, Hoeffel et al.^27^ have shown that in mice TAFA4, a chemokine-like protein that is secreted by sensory neurons, shifts dermal macrophages toward a phenotype that promotes tissue healing. And, Kambrun et al.^30^ and Yoo et al.^28^ showed that in mice C-LTMRs secrete TAFA4, which promotes microglial process retraction and results in decreased production of microglial mediators known to alleviate mechanical pain hypersensitivity. As *TAFA4* is being evaluated for use in a clinical setting^28^, it is crucial to determine which neuronal populations in humans express *TAFA4* and how these map to their preclinical counterparts—such data would inform therapeutic development related to this signaling molecule.

In this paper, we (1) describe a protocol for efficient isolation of DRG nuclei from multiple species, (2) provide the high-resolution, comprehensive, detailed single-nucleus transcriptome atlas of DRG from pre-clinical to human samples, and (3) characterize the transcriptional convergence and divergence of sensory neuron subtypes from rodents to humans. Our results reveal that DRG sensory neuron subtypes are in general well-conserved across species. However, we identified key differences in gene products involved in pathophysiological processes which point to the potential for species-specific sensory neuron functions. Understanding the molecular and functional similarities and differences between somatosensory neurons in rodents and primates will enable a better understanding of the role of these neurons in sensory perception and tissue homeostasis, facilitating therapeutic efforts targeting sensory neurons.

## Results

### DRG neuron enrichment using density-gradient

**Fig. 1:**
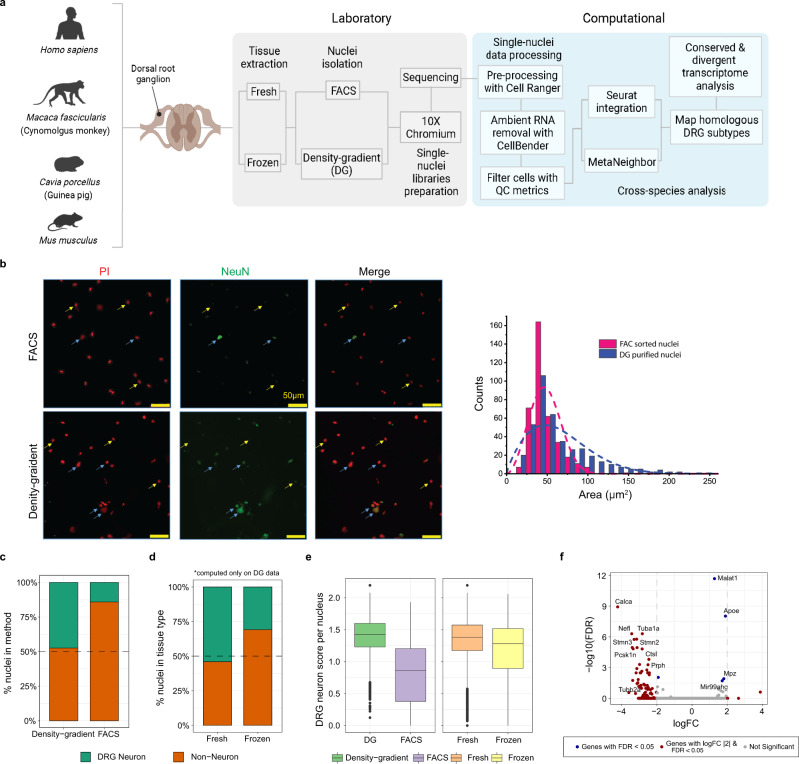
Comparison of nuclei isolation methods and tissue types in dorsal root ganglia single-nucleus RNA-seq. **a** Overview of laboratory and computational workflow. The graphic was created with BioRender.com **b** Representative micrographs of nuclei from FACS and density-gradient (DG) protocols. Blue arrows indicate neuronal nuclei (PI+ and NeuN+) and yellow arrows indicate non-neuronal nuclei (PI+ and NeuN−). Distribution of nuclei size by capture method. Dotted lines represent smoothed distributions of binned data. The experiment was repeated 4 times independently with similar results. **c–e** Summary metrics computed for different nuclei isolation methods and tissue types in mouse data. **c**, **d** Relative cell-type composition for the nuclei isolation methods or tissue types. Black dashed line was drawn at 50%. **e** DRG neuron signature score for different nuclei isolation methods and tissue types. *n* = 37,384 nuclei examined over 10 independent experiments. The lower and upper hinges of the boxes correspond to the 25th–75th percentile with the line in the middle depicting the median. The whiskers are set at the minimum and maximum values of the dataset. **f** Volcano plot showing results from differential expression analysis of transcripts from FACS versus DG nuclei. Statistically significant genes are indicated by red or blue colors. Gray dotted lines represent log-fold change thresholds of 2 and −2.

To characterize the transcriptome of preclinical and clinical DRG samples, we harvested DRGs from 5 mice, 2 guinea pigs, 3 cynomolgus monkeys, and 7 human donors (Fig. 1a, Supplementary Data 1). For all preclinical species, both fresh and frozen tissues were collected, whereas only frozen samples were obtained for human specimens (Fig. 1a, Supplementary Data 1). From these DRGs, we isolated nuclei using two different protocols: a FACS-based method, and a density-gradient (DG) centrifugation method (Fig. 1a). Analysis of nuclei isolated from the FACS protocol revealed that these samples contained fewer larger nuclei, which could suggest a loss of neuronal nuclei during the sorting process (Fig. 1b). We then merged data generated from different isolation methods and tissue types (i.e., fresh and frozen) using the anchoring-based integration approach and clustered the cells using the graph-based clustering approach implemented in Seurat (see the “Methods” section, Fig. 1a).

Comparison of relative cell-type composition between the two isolation methods from mouse DRG samples revealed that the DG protocol captured more DRG neurons than the FACS method (Fig. 1c, Supplementary Fig. 1a). We also observed a similar enrichment of DRG neurons in data from both fresh and frozen tissue samples (Fig. 1d, Supplementary Fig. 1b). Regardless of the nuclei isolation method or the type of tissue sample (fresh or frozen), we detected more transcripts and genes in neuronal nuclei than non-neuronal nuclei (Supplementary Fig. 1c). To further characterize the neurons from our single-nuclei data sets, we computed a DRG neuron signature score*,* reflecting the mean expression levels of DRG-specific marker genes curated from previous reports^7,14,16^. The median DRG neuron signature score per nucleus was higher in DG than in FACS data, whereas the median DRG signature scores between fresh and frozen tissues were comparable (Fig. 1e). Finally, to determine if there were protocol-specific transcriptional differences, we performed differential expression analysis between DG and FACS data. Only 0.2% (41 out of 18545) of expressed genes were statistically differentially expressed between the two methods (Fig. 1f). Within the small number of genes that were differentially expressed, we noticed a slight enrichment of cell type-specific genes that could reflect cell compositional differences resulting from the FACS and DG methods. In general, the FACS and DG protocols produced broadly similar transcription profiles.

Together, these data suggest that the DG nuclei isolation protocol improved our ability to capture and generate the transcriptome data of sensory neurons from either fresh or frozen tissue samples and provide a framework for the isolation and characterization of DRG nuclei from other species.

### Single-nuclei RNA-seq analysis of mouse DRG

**Fig. 2:**
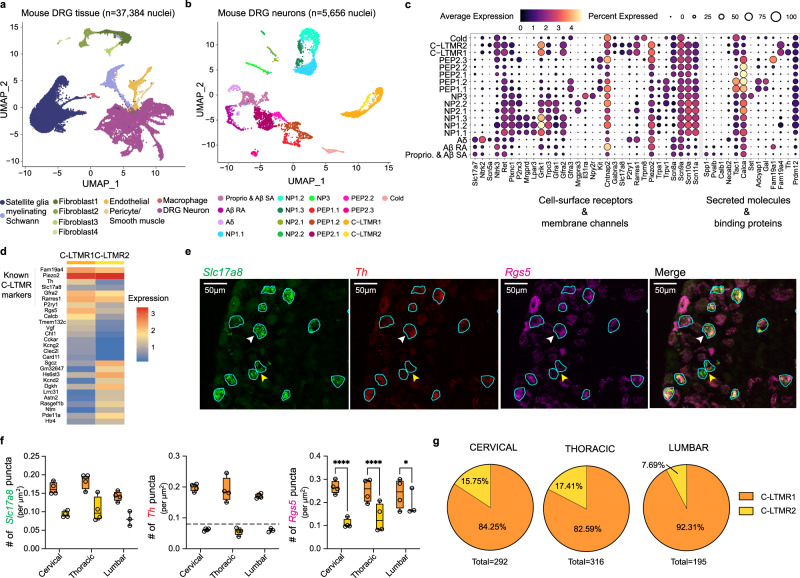
Single-nuclei transcriptome atlas of mouse dorsal root ganglia (DRG) sensory neurons. **a** UMAP of 37,384 mice DRG nuclei colored by cell types and annotated by marker genes as indicated in the main text. **b** UMAP of 5656 mice DRG neurons colored by subtypes and annotated by marker genes as indicated in the main text. NP denotes non-peptidergic; PEP denotes peptidergic for **a** and **b**. **c** Fraction of nuclei (dot size) in each subset expressing canonical marker genes (columns) and their scaled average expression level in expressing cells (dot color) within subtypes (rows). **d** Heatmap comparing mean expression levels (color bars) of top differentially expressed genes (rows) between C-LTMR1 and C-LTMR2 subtypes. **e** Representative images for RNAScope validation of *Rgs5, Th*, and *Slc17a8* expression in mouse DRGs. Neurons are outlined in turquoise. White arrowheads indicate *Slc17a8*-positive cells expressing both high levels of *Th* and *Rgs5* and yellow arrowheads indicate cells that express *Slc17a8*-positive cells but low amounts of *Th* and *Rgs5* transcript. **f** Quantification of RNA transcript punctate dots (representing expression level) normalized by slide area for each DRG level and C-LTMR subtype. *Rgs5* area-normalized puncta per cell differences analyzed using a mixed-effects model (main effect of C-LTMR type: *F*_(1, 8)_ = 175.5, *p* < 0.0001; C-LTMR type and spinal level interaction: *F*_(2, 8)_ = 9.565, *p* = 0.0076) with a Bonferonni’s multiple comparisons post-test C-LTMR1 vs. 2 (cervical & thoracic: adjusted *p* <0.0001; lumbar: adjusted *p* = 0.0131). *N* = 4 mice. Asterisks represent statistical significance (**** for <0.0001 and * for <0.05). Black dashed line represents the manually determined threshold for *Th* high and low cells. The lower and upper hinges of the boxes correspond to the 25th to 75th percentile with the line in the middle depicting the median. The whiskers are set at the minimum and maximum values of the dataset. **g** Distribution of the two C-LTMRs subpopulations by DRG level. Source data are provided as a Source Data file. **f** and **g** share the same color legend.

We integrated expression data from 37,384 nuclei from five mouse DRGs (Table 1, Supplementary Data 1). After clustering, we annotated each cluster with canonical markers (Fig. 2a, Supplementary Data 1; see the “Methods” section). To provide better resolution on the sensory neuron population, we removed clusters containing non-neuronal nuclei and performed iterative clustering on the remaining presumptive neuronal nuclei. Using known mouse DRG sensory neuron subtype-specific markers (Fig. 2b; see also refs. ^7,8,14–16^), we identified 17 transcriptionally distinct DRG sensory neuron subtypes that differ in expression of both cell-surface and secreted molecules (Fig. 2b, c).

Within our mouse data, we identified an additional cluster of C-LTMR neurons (Fig. 2b, c) across all mouse samples (Supplementary Fig. 2a). The two C-LTMR clusters (C-LTMR1 and C-LTMR2) express *Slc17a8* (*Vglut3*), but differ in expression intensity of other known C-LTMR specific markers including *Th, Fam19a4, Rarres1, P2ry1*, and *Gfra2* (Fig. 2d). Renthal et al.^7^ also noted two C-LTMR subtypes that differ in *Th* expression^7^. We performed differential expression analysis of the two C-LTMR populations (Fig. 2d) and identified that genes more highly expressed in C-LTMR2 are associated with signaling pathways and axon guidance gene ontology terms (Supplementary Fig. 2b). In situ hybridization confirmed that C-LTMRs could be divided into two groups with differing *Th* expression, regardless of vertebral level, highlighting the utility of this marker to subset these two subtypes (Fig. 2e, f). Additionally, the expression of *Rgs5*, a regulator of the G-protein signaling family, is correlated with high expression of *Th* in C-LTMR1 neurons, regardless of the level of the DRG. While we did not observe any differences in the cell diameter size of these two populations of C-LTMRs (Supplementary Fig. 2c), we did find that the proportion of C-LTMR subtypes differed by DRG levels, with lumbar having the fewest C-LTMR2 (7.69% in lumbar versus 17.41% in thoracic and 15.75% in cervical; 4 biological replicates per level; Fig. 2g).

Overall, our single-nuclei expression data identified the major known sensory neuron populations in the mouse DRG. Additionally, a second subpopulation of transcriptionally distinct C-LTMR sensory neurons was identified that differ in their proportion across DRG levels, which could reflect that these neurons transduce distinct sensory information.

### Cross-species mapping of DRG sensory neuron subtypes

**Fig. 3:**
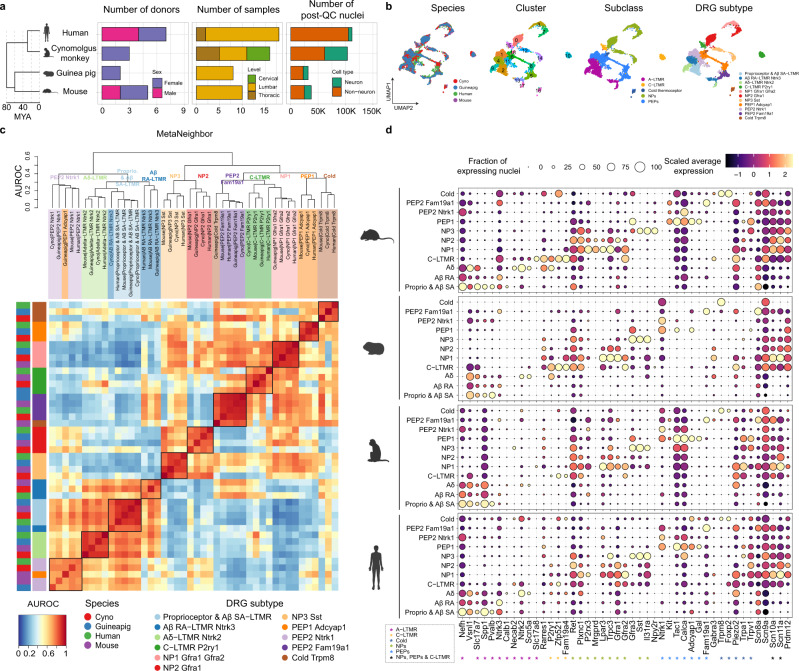
DRG sensory neuron homology consensus across mouse, guinea pig, cynomolgus monkey and human. **a** Summary metrics for mouse, guinea pig, cynomolgus monkey, and human DRG single-nuclei RNA-seq data. Evolutionary distances (Million Years Ago; MYA) are shown in the dendrogram to the left. **b** UMAP of Seurat-integrated DRG neurons across mouse, guinea pig, cynomolgus monkey, and human, faceted and colored by species, Seurat clusters, DRG subclasses, and subtypes. **c** Cross-species dendrogram and heatmap of MetaNeighbor mean area under the receiver operator characteristic (AUROC) scores (*y*-axis in plot) colored by DRG sensory neuron subclasses. **d** Fraction of nuclei (dot size) in each neuron subtype expressing selected canonical markers (columns) and their scaled average expression level in expressing nuclei (dot color) in mouse, guinea pig, cynomolgus monkey, and human data, by sensory neuron subtypes (rows). Colored asterisks indicate markers used for annotating clusters in the integrated cross-species DRG data. NP denotes non-peptidergic; PEP denotes peptidergic for **b**–**d**. Species icons in **a** and **d** were created with BioRender.com.

To further expand our understanding of somatosensory function in non-mouse species, we performed single nucleus RNAseq analysis of DRGs from guinea pig, cynomolgus monkey, and human samples (Fig. 3a, Table 1, Supplementary Fig. 3a, Supplementary Data 1). In our human data, the number of nuclei varied across donors and samples (Supplementary Fig. 3b). Most of our human donors are males (5 male and 2 female) with ages varying from 21 to 48 years old (Supplementary Data 1). All cell types were seen across all donors, although the majority of adipocytes were isolated from a single female donor (Supplementary Fig. 3b). H&E imaging of the tissues suggests that while the neuron size increases from mouse to cynomolgus monkey to human, we did not notice an appreciable difference in the size of satellite glia (Supplementary Fig. 3c).

To map DRG sensory neuron populations across species, we performed comparative analysis using two different methods, Seurat and MetaNeighbor (see the “Methods” section). We used Seurat for aggregating our cross-species datasets using anchors identified from canonical correlation analysis^17^ and MetaNeighbor for quantifying cell-type replicability in cross-species datasets^18^. Previous cross-species studies^19–21^ demonstrated that both Seurat and MetaNeighbor provide effective computational methods to map cell types across independent datasets to reveal cell-type relationships among species. Using Seurat, we integrated DRG sensory neuron subsets across species (Fig. 3b). Sensory neuron nuclei were well-integrated between different species and clustered according to their subtype identities, rather than by species. (Fig. 3b, Supplementary Fig. 4b). In parallel to our approach using Seurat, we also performed MetaNeighbor analysis on the DRG neuron subsets. The dendrogram of the mean area under the receiver operator characteristic (AUROC) curve scores was organized by the major DRG subtype category rather than by species (Fig. 3c, Supplementary Fig. 4a). Together, the results of these analyses, derived from two distinct computational approaches, are well-aligned, highlighting that sensory neuron subtypes are generally well-conserved across species. Our computational strategy enabled cross-species, high-resolution DRG cell type annotation, providing an interpretable comparison of gene expression profiles from preclinical models to humans.

Leveraging our well-aligned cross-species data, we compared the expression patterns across species of genes known for their roles in sensory function (Fig. 3d). These data highlight known gene expression profiles, such as the ubiquitous expression of *Scn9a* (Na_v_1.7) across all sensory neuron subtypes. Our data also show that *Scn10a* (Na_v_1.8) is primarily expressed in NPs, PEPs, and C-LTMRs across all species, and *Calca* is more broadly expressed in primates (both NPs and PEPs) while it’s more specifically expressed in PEPs in mice. Additionally, our data highlight a number of other cell-type-specific expression patterns. For example, proprioceptors and A$\beta$ SA-LTMRs express known markers including *Pvalb, Ntrk2, Ntrk3*, and *Slc17a7*; A$\beta$RA-LTMRs express high levels of *Ntrk3, Slc17a7*, and *Vsnl1*; and A$\delta$-LTMRs distinctively express *Ntrk2* and *Scn5a*; C-LTMRs express *Gfra2, Piezo2*, and *P2ry1* along with the newly identified C-LTMR-specific marker^16^, *Zpf521/ZNF521*; NP1 express *Gfra1, Gfra2, Trpc3*, and *Plxnc1*; cold thermoreceptors express *Trpm8, and Foxp2* but do not express *Piezo2*;NP2 express *Gfra1, Trpc3*, and *Plxnc1*, but are negative for *Gfra2*; and NP3 express *Sst* and *Il31ra*; PEP1 express *Gal, Adcyap1*, and *Trpa1*; and PEP2 Ntrk1 express *Ntrk1, Tac1, Calca*, and *Nefh*; PEP2 Fam19a1 express *Fam19a1, Piezo2*, and *Kit*.

We also compared our data to other recently generated DRG expression data^14–16,31,32^. For example, we used marker genes defined by Zeisel et al.^32^ and Sharma et al.^31^ to map our PEP clusters to the peptidergic/CGRP clusters published in these studies(Supplementary Fig. 5a). Additionally, we used markers used from Kupari et al.^16^ to map DRG subtypes from rhesus macaque to our cynomolgus monkey data. These clusters aligned well, with the exception of A-LTMRs for which our data captured different subpopulations (Supplementary Fig. 5b). When we compared our human data with recent single-nuclei RNA-seq data from Nguyen et al.^14^ and spatial transcriptome data from Tavares-Ferreira et al.^15^, we observed differences in subtype classification across these data sets (Supplementary Fig. 5b). For example, the H10 cluster in Ngyuen et al.^14^ and pruritogen enriched receptor cluster in Tavares-Ferreira et al.^15^ map to both NP1 and NP2 in our data. Additionally, while Tavares-Ferreira et al.^15^ noted that they did not observe known C-LTMR-specific markers including *TAFA4, P2RY1*, and *Zpf521/ZNF521* expression in their putative C-LTMR, we observed these markers’ expression in our human C-LTMR (Fig. 3d). While these other data provide additional insights around the transcriptional landscape of human DRGs, the inconsistent DRG subtype nomenclature across studies makes it challenging to compare and interpret gene expression profiles from these datasets to those generated from preclinical models. Our approach produced a well-integrated, cross-species atlas with harmonized DRG subtype classification that enables comparative analysis of sensory neurons.

### Comparative analysis of DRG sensory neuron transcriptomes

**Fig. 4:**
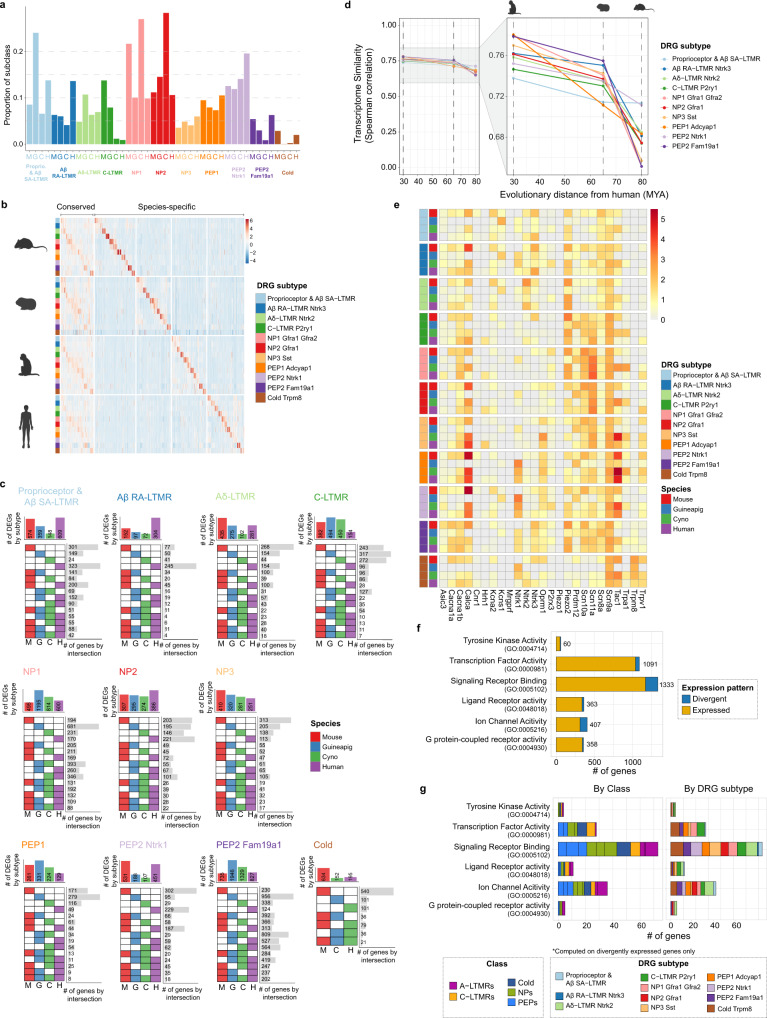
Conserved and species-specific transcriptional programs in DRG sensory neurons. **a** Relative DRG subtype proportions across all species (computed in lumbar levels only). **b** Heatmap of conserved genes and species-specific genes by DRG subtypes from panel (**c**). **c** Customized UpSet plot showing a number of conserved and species-specific genes across datasets for each of the major sensory neuron subtypes, as indicated above each plot. Total number of genes that are unique to one species (one colored box per row), or shared across multiple species (multiple colored boxes per row), are indicated to the right of each row. **d** Plot indicating evolutionary distance (*x*-axis; icons at top) and transcriptional correlation (*y*-axis) for each sensory neuron subtype (indicated by colors) between humans and other preclinical species. Correlation values are indicated by colored circles; the values across all species are connected with a solid line. **e** Heatmap indicating normalized expression levels of genes associated with pain perception in each neuronal subtype. **f** Number of genes with a divergent or non-divergent expression between mouse and human DRGs indicated by gene ontology(GO) annotation category. Numeric numbers on each bar represent the total number of expressed genes in a given GO category. **g** Number of divergently expressed genes between mouse and human samples from panel (**f**), colored by DRG class (left) or subtype (right). NP denotes non-peptidergic; PEP denotes peptidergic for (**a**–**g**). M denotes mouse, G denotes guinea pig, C denotes cynomolgus monkey, and H denotes human for (**a**–**c**). Species icons in **b** and **d** were created with BioRender.com.

Leveraging the integrated single-nuclei data, we performed analyses to assess the similarities and differences of sensory neurons across species. First, we compared the relative proportions of sensory neurons across species by calculating the frequency of each subtype within individual species (analysis restricted to lumbar level DRGs; Fig. 4a). We observed a smaller proportion of C-LTMRs in cynomolgus monkey and human (~1% and ~0.8%, respectively), as compared to the guinea pig and mouse (8% and 14%, respectively). Notably, we observed that peptidergic neurons (PEP1, PEP2 Ntrk1, and PEP2 Fam19a1) comprise a majority of the sensory neuron populations from human DRGs within our analysis. To validate the C-LTMR proportion differences that we observed across species, we performed in-situ hybridization using probes specific for C-LTMRs (Supplementary Fig. 6a–c). We found similar proportions of C-LTMRs in rodents using in-situ hybridization compared with our single-nucleus data. Conversely, we detected a higher proportion of C-LTMRs in cynomolgus monkeys and were unable to detect C-LTMRs in human DRG sections with in-situ hybridization. These data highlight potential technical differences in DRG neuron subtype detection across species using single-nucleus sequencing technologies or 2D FISH analysis.

For subtype-specific genes conserved across all four species, we performed gene ontology analysis to assess conserved biological programs (Fig. 4b, c, Supplementary Fig. 7a, Supplementary Data 2, see the “Methods” section). Notably, this analysis revealed the enrichment of distinct biological pathways across sensory neuron subtypes which reflect that a variety of cellular pathways are utilized differentially by distinct subtypes to transduce sensory information. For example, A$\beta$ RA-LTMR subtype, which is identified by expression of *Ntrk3, Scn1a*, and *Atp2b2*, is enriched for genes associated with action potential conduction and rapid response to mechanical stimuli and could point to biological programs most relevant for sensing differences in touch sensation^33^. Additionally*,* the PEP1 subtype, which is identified by the expression of *Adcyap1, Trpv1, Tac1*, and *Oprm1*, is enriched for genes associated with acute inflammatory response and the perception and modulation of pain signaling. The PEP1 subtype could be useful in the identification of conserved neuro-immune signaling pathways, which might also be relevant for therapeutic development.

We also examined correlations between the transcriptomes of species pairs to gain further insight into the evolutionary divergence of sensory neuron subtypes (Fig. 4d, Supplementary Fig. 8b). This highlighted that cynomolgus monkeys and humans share the highest correlation between transcriptomes across all subtypes. Our analysis further revealed that transcriptomes of Proprioceptors and A$\beta$ SA-LTMRs are the most correlated between humans and mice (*⍴* = 0.71), while the transcriptomes of the NP1 and PEP2 Fam19a1 subtypes (*⍴* = 0.65 and 0.65, respectively) are the least correlated between mouse and human (Supplementary Fig. 8a).

Finally, we examined the expression of a set of genes including ion channels, ligand-gated channels, G-protein-coupled receptors (GPCRs), and neuropeptides that have been well-studied for a role in sensory transduction and could provide therapeutic targets (Fig. 4e, Supplementary Fig. 9a–f). From this analysis, we observed divergent expression patterns of these sensory molecular determinants across species. For example, analysis of genes involved in pain perception (Fig. 4e) highlighted that *TRPV1* was expressed in more broader sets of subtypes including C-LTMRs, all NPs, all PEPs and cold thermoreceptors in human DRGs, while *Trpv1* expression was restricted to subsets of NPs, PEPs, and cold thermoreceptors in mouse, guinea pig and cynomolgus monkey DRGs. Additionally, *SCN8A* was expressed in essentially all DRG subtypes in primates whereas *Scn8a* was selectively expressed in LTMRs and PEPs in mice.

Leveraging these data from our harmonized cross-species atlas enabled detailed interrogation of individual genes and gene sets from preclinical models to humans, and pointed to divergent and conserved biological processes. Further, these data can help inform therapeutic programs targeting sensory neuron-mediated pathophysiological processes such as pain perception, and can also help inform our understanding of any species-specific adaptations that have evolved.

### Divergent expression of TAFA4 across species

We were interested in performing a broad survey of the expression of genes associated with the druggable genome. For this, we collected the list of genes annotated with the ontology terms ion channels, G protein-coupled receptor, tyrosine kinase, ligands, and signaling-pathway-related molecules. We assessed the expression of these genes across preclinical and human samples and identified those genes that displayed divergent expression patterns between mice and humans (Fig. 4f). Interestingly, within the “Signaling Receptor Binding” category we observed that 18% (235) of the 1333 genes were expressed in distinct subtypes of DRG neurons between mouse and human. Of note, the sensory neurons with the largest number of divergent genes across all druggable genome GO terms were the nociceptors (PEP and NP; Fig. 4g).

**Fig. 5:**
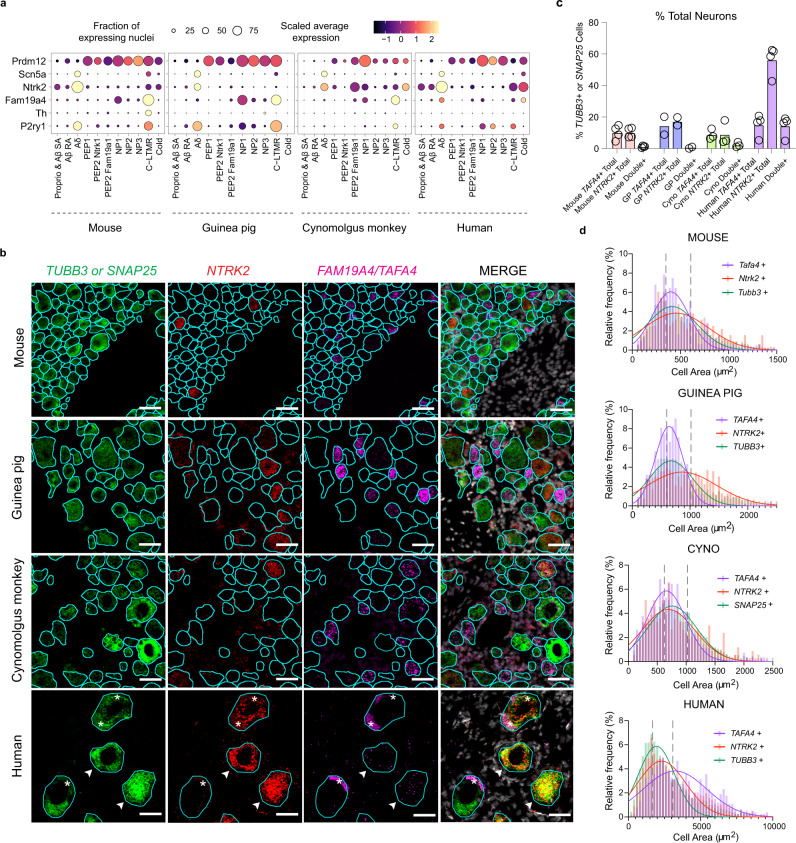
Divergent expression of TAFA4 across species. **a** Dot plots showing the fraction of nuclei (size) and their scaled average expression level (color) in each neuron subtype (columns) across mouse, guinea pig, cynomolgus monkey, and human for genes (row names). NP denotes non-peptidergic; PEP denotes peptidergic. **b** Representative images of RNAScope validation of *FAM19A4/TAFA4, NTRK2*, and *TUBB3* (mouse, guinea pig, human) or *SNAP25* (cynomolgus monkey) in DRGs. Neurons are outlined in turquoise. White arrowheads indicate neurons (*TUBB3*+ or *SNAP25*+) expressing both *TAFA4* and *NTRK2* in human samples. Asterisks in the human images represent lipofuscin aggregates. Scale bar represents 50 µm. GP denotes guinea pig; Cyno denotes cynomolgus monkey. **c** Percentage of *FAM19A4/TAFA4*+, *NTRK2*+, and *TAFA4+/NTRK2+* (‘double +’) in mouse, guinea pig, cynomolgus monkey, and human. **d** Size distribution of *FAM19A4/TAFA4+, NTRK2+*, and *TUBB3+* or *SNAP25+* cells in each species. Colored solid lines represent smoothed distributions of binned data. Gray dashed lines represent the 33% and 67% percentiles of the *TUBB3+* or *SNAP25+* distribution as a surrogate for small, medium, and large diameter neurons. Source data are provided as a Source Data file.

One gene of interest from the “Signaling Receptor Binding” category is *Tafa4/Fam19a4* which is expressed by sensory neurons and has been shown to play a role in the maintenance of tissue homeostasis by modulating the function of Il10+ dermal macrophages and microglia^27,28,30^. In our mouse data, *Tafa4* is predominantly expressed by C-LTMRs and is also expressed by some NPs (Fig. 5a). Notably, the expression of *TAFA4* in our human data is distinct from the preclinical models as *TAFA4* is most strongly expressed in A$\delta$-LTMRs that co-express *NTRK2* and *SCN5A*, but also expressed in cold sensing neurons and C-LTMRs (Fig. 5a). Additionally, we observed the same divergent expression pattern of *TAFA4* in trigeminal ganglia^34^ (Supplementary Fig. 5c). To validate these findings, we performed in situ hybridization (ISH) to assess the expression of *TAFA4* transcripts in *NTRK2*+ neurons. Consistent with our single nuclei RNAseq data, FISH images revealed that ~98% of *TAFA4*+ neurons are *NTRK2*+ in human DRGs whereas, in DRGs from other species, we observed very few cells with expression of both markers (~9% in mouse, ~1.3% in GP and ~9% in Cyno; compare ‘TAFA4+’ to ‘Double+’ across species; Fig. 5b, c). A$\delta$ fibers are characterized as having a larger cell body diameter than C fibers. Therefore, to determine if the expression of *NTRK2* and *TAFA4* was observed more frequently in larger diameter neurons in human samples as would be predicted from our data, we assessed the average cell body area of neurons across all species that express *TAFA4* and *NTRK2*. Consistent with our single nuclei RNAseq and in situ data, we observed that the cell area of *TAFA4*+ neurons in mice, guinea pigs, and cynomolgus monkeys predominantly fell into the small-to-medium range whereas in human samples, this cell area distribution shifts towards larger neurons (Fig. 5d). Together these data indicate significant differences in the cell-type expression of *TAFA4* across species, which will have implications for therapeutic strategies targeting TAFA4.

Taken together, this analysis highlights that while DRG subtypes are, in general, conserved from pre-clinical models to humans, significant differences exist between species. Understanding the transcriptional and molecular differences across species will be critical to enabling a more complete understanding of pain and other pathophysiological processes, and for the development of effective therapeutics. Importantly, these data amplify the need for accurate, detailed, and well-controlled cross-species atlases to more fully understand similarities and differences between preclinical and human sensory neuron function.

## Discussion

Comparative cross-species analysis using atlases of single nuclei data is a powerful approach that can provide a high-level view of the cellular composition of tissues and a detailed map of the molecular landscape of these tissues. Such analyses can inform our understanding of biological mechanisms within tissues and can provide perspective on how these mechanisms might have evolved across species. Importantly, these data will play a central role in informing the translation of data from rodents to primate models and humans. Our framework can improve the capture of sensory neuron nuclei even in frozen tissues and will likely translate well for generated atlases of other sensory neurons such as those from the nodose ganglia. Our integrative and comparative analysis of DRGs from mice, guinea pigs, cynomolgus monkeys, and humans identified diverse subtypes of DRG sensory neurons in each species and showed that these subtypes are generally conserved. Our analysis highlighted conserved transcriptional features which may reflect core biological mechanisms that are relevant for subtype-specific sensory functions. Additionally, we identified divergent expressions of molecular determinants involved in sensory function which highlight species-specific adaptations for pathophysiological processes like pain and tissue repair. Among those divergently expressed genes, we validated *TAFA4*, a known key modulator of the neuro-immune axis. *TAFA4* is expressed in distinct populations of sensory neurons across mouse and human DRGs, which is relevant for therapeutic strategies targeting TAFA4. This paper provides a comprehensive, harmonized, and interpretable cross-species atlas and analysis of sensory neurons that will be crucial for a better understanding of sensory neuron function and will enable the development of more effective pain and neuro-immune therapeutic targets.

To fully leverage these data, we explored different integrative computational approaches to align homologous sensory neuron subtypes across species and build a comprehensive cross-species atlas from preclinical models to humans. These approaches included Seurat to aggregate datasets, and MetaNeighbor to identify cell types that are highly replicated among datasets. Each of these methods has distinct caveats. For example, the data aggregation algorithm implemented in Seurat can be susceptible to over-integration when only a subset of the cell populations is preserved among datasets. In addition, MetaNeighbor was developed to describe the extent of cell-type reproducibility across scRNA-seq data sets, rather than quantitatively classifying cell types between query and reference data sets. However, in our analysis, the cell-type homology mapping results from Seurat and MetaNeighbor were well-aligned, suggesting that these results are robust, providing a computational framework for more systematically performing a such cross-species comparison. Our computational work yielded a comprehensive cross-species atlas that described 11 transcriptionally distinct DRG sensory neuron subtypes across four species, using common subtype nomenclature. Future analysis using high-resolution spatial technologies such as MERFISH^35^, or methods to characterize cell-type specific electrophysiological activity such as Patch-seq^36^, would provide a more complete picture of sensory DRG neurons across species.

Although our data captured sensory neuron subtypes that are similarly described in the recently published analysis of primate DRGs data^14–16^, we noted some significant differences in the resolution of subtype classification and molecular marker expression which are relevant for interrogating sensory mechanisms from pre-clinical models to human. These differences likely reflect a combination of factors including the number of cells sequenced, the resolution of the sequencing technology that was used, or the data integration framework used in the analysis, which can all have an impact on sensory neuron subtype annotation. The data on their own cannot be used off-the-shelf, but as we have shown, require careful integrative analysis that takes into account technical or biological differences between datasets.

Leveraging our cross-species atlas data, we identified differences in the proportion of types of sensory neurons (i.e. C-LTMRs or NPs) across species. These differences in proportion could reflect differences in biology across the species or levels of DRGs. For example, differences in the C-LTMRs may reflect species-specific differences in the perception of specific cues, including those related to glabrous and hairy skin, or perception of itch. It is notable that we find a well-characterized C-LTMR marker for mice, *FAM19A4*, expressed in *TRPM8*+ and A$\delta$-LTMR neurons. It would be interesting to know whether these human *TRPM8*+ and A$\delta$-LTMR neurons perform a similar function as mouse C-LTMRs in modulating pain sensations. Our data show that mouse C-LTMRs, human *TRPM8*+ neurons, and human A$\delta$-LTMRs express the ion channels *KCNJ6 (GRIK2)* and *CCDC109B* (mitochondrial calcium uniporter dominant negative beta subunit), suggesting a common mechanism for modulating calcium homeostasis and excitability. It would be interesting to explore the functional implications of these similarities using electrophysiology or functional imaging in the future. Together, these data help identify different biological mechanisms that will ultimately inform therapeutic development targeting sensory neurons.

We performed additional analyses to assess transcriptional features reflecting core sensory neuron functions or species-specific adaptations. Interestingly, in the context of their gene expression profiles across species, we found that Proprioceptors and A$\beta$ SA-LTMRs are the most conserved sensory neuron subtypes whereas NP1 and PEP2 Fam19a1 were the least conserved. This would be consistent with the important role that proprioceptors play in sensing space and limb positioning or enabling fast muscle reflexes, all of which are crucial for vertebrate survival. Whereas sensations perceived by other sensory neuron subtypes are more tuned to an animal’s immediate environment and are manifest in gene expression variability within these subtypes. Differential expression analysis highlighted conserved gene expression programs that could reflect the distinct cell-type-specific sensory function. In particular, the identification of conserved genes involved in pain signaling and acute inflammatory response among the peptidergic nociceptors present opportunistic therapeutic targets and inform our understanding of clinical programs targeting sensory neuron function.

Our analysis also highlights widespread expression differences of peptide neurotransmitters between rodent and primate DRG neurons. The functional role of these peptide transmitters should be further investigated as these differences may have relevance to pain sensation between rodents and humans. Of interest, CGRP immunoreactivity in the human dorsal spinal cord was recently reported to be restricted to, but seen throughout, the superficial laminae within the substantia gelatinosa^37^. This contrasts with findings in mice of restricted CGRP immunoreactivity in lamina I and II outer of the dorsal horn^38–41^. While peptidergic input to the primate spinal cord may be more spatially diverse, it still remains unclear whether distinct transcriptionally defined cell types defined across species (e.g. PEP1, PEP2, NP3) retain their input patterns into the spinal cord, and potentially their functional distinctions.

We used our atlas to investigate genes relevant to sensory function and pathophysiological processes (e.g. receptors and signaling molecules) to determine whether they might be functionally conserved across species. Our analysis highlights that these genes were the most diverged in their expression patterns across species, especially within the nociceptors. This may have implications for understanding the evolution of sensory responses and could improve drug discovery efforts targeting nociceptors for pain. An intriguing example from this analysis was *TAFA4/FAM19A4*, a chemokine-like protein known to be secreted by GINIP+ DRG neurons in rodents that have been studied for its role in chronic pain and tissue repair. Both our expression data and FISH analysis confirmed the differential expression of *TAFA4/FAM19A4* in human DRGs. It will be interesting to determine if the functional neuro-immune signaling axis mediated by TAFA4/FAM19A4 is conserved in humans, even though *TAFA4/FAM19A4* may not be produced by the same subsets of sensory neurons. Future work exploring the role of TAFA4/FAM19A4 in pain or tissue repair could provide insights that inform efforts to translate therapeutic targets from animal models to the clinic.

The strength of the work presented here includes the development and use of a consistent laboratory protocol to avoid technical artifacts that can occur during single-cell sequencing; the generation of single nuclei data representing a broad sampling of sensory neurons across multiple species; the application of multiple computational approaches to provide confident and detailed cell-type mappings across species; and harmonized nomenclature of sensory neuron subtypes from mouse, guinea pig, cynomolgus monkey, and human. The appropriate pairing of laboratory methodologies and computational approaches can have a significant impact on the development and ultimate downstream utility of cross-species atlases. Our ability to identify, characterize, and validate the differential expression of *TAFA4* across species, highlights a significant example of the utility of our approach both in our ability to understand the neuro-immune compartments and to develop clinically relevant therapeutics. Our analysis provides a more complete understanding of the general principles driving somatosensory mechanisms and informs our understanding of the role of sensory neurons in the maintenance of tissue homeostasis. Collectively, this work enables the development of more effective therapeutics targeting sensory neuron function.

## ‘Methods’

### Animals

Care and handling procedures of animals were reviewed and approved by the Genentech Institutional Animal Care and Use Committee (IACUC) and animal experiments were conducted in full compliance with IACUC policies and NIH guidelines.

Mice used in this study were C57BL/6J (The Jackson Laboratory, Stock No. 007914). Mice used in this study were 6–17 weeks old and both female and male mice were used. Hartley guinea pigs (Charles River Laboratory) used in this study were 5.5–6 months old and only female guinea pigs were used. The laboratory mice were kept on a 12-h light/dark cycle at the controlled room temperature of 20–22 °C with humidity of 40–50% for the duration of the experiments.

### Tissues

Cynomolgus monkey DRGs from three animals were purchased from Covance in two shipments, Fresh (isolated, placed in Hibernate A solution (BrainBits, Catalog #HALF500), delivered at 4 °C overnight) and Frozen (flash frozen at isolation, delivered on dry ice). The age of the animals varied from 8 to 9 years and only female monkeys were used.

Frozen Human DRGs were obtained from Anabios (6 donors) and Donor Network West (1 donor; 1 pair of lumbar 4 level). The DRG tissues from Anabios were stored in liquid nitrogen prior to shipment. All samples were assessed for tissue integrity prior to downstream applications.

All human tissue samples were supplied by AnaBios Corporation and Donor Network West. Each supplier received IRB approval of research, appropriate informed consent of all subjects contributing biological materials, and all other authorizations, consents, or permissions as necessary for the transfer and use of the biological materials for research at Genentech. Patients/human donors were not compensated for their donation of tissues.

Additional details on individual animals and samples are available in Supplementary Data 1.

### Tissue processing

Mice were euthanized by CO_2_ inhalation and decapitation. DRGs from lumbar 1–6 levels from both right and left sides were extracted, de-sheathed, and placed in 1 mL ice-cold lysis buffer (20 mM NaCl, 5 mM MgCl_2_, 0.1% TX-100, 10 mM Tris–HCl pH 7.2) containing EDTA-Free protease inhibitor (Sigma, Catalog #4693124001), RNAse inhibitor 0.2 U/mL (Life Technologies, Catalog # N8080119), and RNase-free DNase (Promega Corporation, Catalog #M6101), in a 2 mL Dounce homogenizer tube (Kimble Chase, Catalog #885300-0002).

Guinea pigs were euthanized using protocols approved by the Genentech Institutional Care and Use Committee. Briefly, the animals were placed in an isoflurane chamber for anesthesia. Once anesthetized, the animals were injected with a 1 mL solution of Euthasol and Sterile saline in a 1:1 ratio. Death was confirmed by loss of heartbeat and corneal opacity before decapitation and DRG dissection. DRGs from lumbar 3–5 levels from both right and left sides were collected and placed in the lysis buffer described above.

DRG samples from cynomolgus monkeys and humans were first cut into ~1–2 mm pieces with a scalpel while placed on a dissection tray over dry ice and then placed in the lysis buffer described above.

### Sensory neuron nuclei isolation

Dounce homogenization was used to dissociate all DRG tissues. Before douncing, 1 mL HBSS (Thermo Fisher, Catalog #14025092) containing 3% BSA Fraction VI and RNAse inhibitor (nuclei suspension buffer, NSB) was added to the 2 mL Dounce Tissue Grinder (Kimble Chase, Catalog #885300-0002). The DRGs were homogenized with an A (“loose”) pestle using 5–10 strokes. The homogenate was then filtered through a 70-micron filter and spun down. The pellet was resuspended in NSB. The frozen DRGs were not allowed to thaw before placing in the lysis buffer and douncing.

For gradient-purified nuclei, we used an Optiprep density gradient (Sigma, Catalog #D-1556; 35%, 16%, for rodents and 40%, 20%, and for primates) as described below. Optiprep gradients were prepared in HBSS. The filtered homogenate suspended in NSB was mixed with an equal volume of 16% (rodent samples) or 20% (primates samples) optiprep solution and layered carefully over the 16% or 20% optiprep layer. The gradient tubes were centrifuged in a cooled swinging bucket rotor at 2500×*g* for 20 min. Nuclei at the 16/35 interface were aspirated and mixed with an equal volume of NSB. Nuclei were re-pelleted, washed, suspended in NSB, and counted using a hemocytometer (gradient purified) prior to loading into a 10X Genomics Chromium controller.

For FACS nuclei isolation, nuclei from the density gradient were pelleted by centrifugation, labeled with propidium iodide (Life Technologies, Catalog #P1304MP) and DAPI (Life Technologies Catalog #62248) and sorted using a FACSAria Fusion Flow Cytometer (BD Biosciences). Nuclei were selected based on double labeling with DAPI and PI (Supplementary Fig. 10) and sorted into Eppendorf tubes containing 0.5 mL nuclei suspension buffer (NSB, described above). Nuclei were then counted, pelleted, and resuspended into an appropriate volume prior to loading into a 10X Genomics microfluidic chip for droplet generation and barcoding.

### Preparation of single-nuclei RNA-sequencing libraries

Chromium Next GEM Single Cell 3ʹ GEM, Library & Gel Bead Kit v3.1 (10X Genomics PN-1000121) were used for library preparation according to the manufacturer’s user guides. The Cell-RT mix was prepared to aim for 10,000 nuclei per sample and applied to the Chromium^TM^ Controller for GEM generation and barcoding. Then samples were subjected to post-GEM-RT cleanup, cDNA amplification (11 cycles with v3.1), and library construction according to the user manual. Sample index PCR was done with 12 cycles. Libraries were then quantified by Qubit dsDNA HS Assay Kit (Thermo Fisher Q33230) and profiled by Bioanalyzer High Sensitivity DNA kit (Agilent Technologies 5067-4626). Libraries were sequenced by HiSeq4000 (Illumina) following the 10X Genomics sequencing specification.

### Reference genomes

For genomic mapping, we augmented GRCm38, Cavpor3.0, macFas5, and hg19 reference transcriptomes with introns to allow both pre-mRNAs and mature mRNAs to be mapped. GRCm38, Cavpor3.0, macFas5, and hg19 reference transcriptomes were modified according to the instructions provided by the 10X Genomics website (https://support.10xgenomics.com/single-cell-gene-expression/software/pipelines/latest/ advanced/references).

### Preprocessing and alignment of single-nuclei data

Single-cell RNA sequencing data were processed with a CellRanger analysis pipeline. Briefly, reads were demultiplexed based on perfect matches to expected cell barcodes. Transcript reads were aligned to the appropriate species genome using GSNAP (2013-10-10)^42^. Only uniquely mapping reads were considered for downstream analysis. Transcript counts for a given gene were based on the number of unique UMIs for reads (up to one mismatch). Both intronic and exonic reads were used to determine transcript count. Cell barcodes from empty droplets were filtered by requiring a minimum number of detected transcripts. Data quality for individual libraries was assessed based on total read depth, percentage of reads with valid barcodes, percentage of demultiplexed reads in detected cells, number of detected cells, and number of analyzed cells. Sample quality was further assessed based on the distribution of per-cell statistics, such as total number of reads, percentage of reads mapping uniquely to the reference genome, percentage of mapped reads overlapping exons, number of detected transcripts (UMIs), number of detected genes, and percentage of mitochondrial transcripts.

### Removal of background noise in gene expression matrices

We used the ‘remove-background’ function of CellBender (v.0.2.0) to remove technical ambient RNA counts and empty droplets from the gene expression matrices^43^. Cell Ranger-generated ‘raw_feature_ bc_matrix.h5’ files were used as input for CellBender. The parameter ‘expected-cells’ was obtained from the Cell Ranger metric ‘Estimated Number of Cells’, while the parameter ‘total-droplets-included’ was set to a value between 8000 and 12,000 to represent a point within the plateau of the barcode rank plot in all samples.

### Quality control and clustering

After this initial quality control, nuclei with <1000 total UMIs, 500 unique detected genes, and >25% mitochondrial UMIs were discarded. After the filtering step, the gene × cell matrix of raw UMI counts was log-normalized using ‘NormalizeData()’ in SeuratV3^17^ in R (v4.1.1) environment. All libraries within each species were integrated using ‘FindIntegrationAnchors()’ and ‘IntegrateData()’ functions with all default parameters including the dimensionality of each dataset set at 30 in SeuratV3 (see below table for # of dimensions used for each analysis). Then, we scaled the species-specific integrated data, performed dimensionality reduction by PCA, calculated UMAP coordinates and Louvain clustering for all nuclei using SeuratV3 (Figs. 2a, b, 3a–c, Supplementary Fig. 3a–c).

**Table 1:** Canonical correlation (CC) dimensions used in Seurat integration analyses, by cell type and species

Dataset	Cell type	Used CCs
Mouse DRG	All cell types	30
Mouse DRG	Neurons	30
Guinea Pig DRG	All cell types	30
Guinea Pig DRG	Neurons	30
Cynomolgus Monkey DRG	All cell types	30
Cynomolgus Monkey DRG	Neurons	30
Human DRG	All cell types	30
Human DRG	Neurons	30

Major cell type clusters were identified based on top differentially expressed genes from each cluster (Figs. 2a, b,3a–c, Supplementary Fig. 3a–c). In all species-specific data, clustering was performed twice: first, to separate neurons and glia from other cells, and then to sub-cluster the DRG neurons to obtain high-resolution clusters within the DRG neuron group.

### DRG neuron signature score

The DRG neuron signature score reflects the mean expression levels of a set of marker genes for neurons. We compiled the following lists of neuronal marker genes for DRG neurons from the literature^7,14,16^: *Ret, Ldhd, Nefh, Cntnap2, Scn9a, Scn8a, Scn10a, Scn11a, Tac1, Plxnc1, Gfra2, Calca, Slc17a7, S100b, Piezo2, Uchl1, Rbfox3*, and *Snap25*. We calculated the average expression of these genes to construct the “DRG neuron” signature score (Fig. 1f) and used this score to assess neuron nucleus quality across nucleus isolation methods and tissue types.

### Differential expression analysis methods and tissue types

To compare the quality of transcriptomic profiles generated by different isolation methods and sample types, we performed a differential expression (DE) analysis. Pseudo-bulk expression profiles were derived from single-cell datasets by calculating the average of the total number of UMIs for each gene across all nuclei of each sample. This gave a gene-by-pseudobulk count matrix which was then normalized to a normalized count statistic using the ‘calcNormFactors()’ function from edgeR. DE analysis was performed by calling ‘glmLRT()’ and then using ‘topTags()’ to extract the final differential expression statistics.

### DRG neuron subset-specific analysis

To obtain high-resolution clusters within the DRG neuron subset in all species-specific data, we first removed all non-neuronal nuclei barcodes, and then nuclei that express any satellite glial specific transcripts (*Plp1* < 1 & *Mpz* < 1 & *Sparc* < 1) were removed. The resulting digital gene-expression matrix (DGE) was carried forward for clustering.

We annotated different subsets of large diameter myelinated A-LTMRs using *Nefh, Slc17a7, Pvalb, Spp1, Calb1, Ntrk3, Scn5a, Ntrk2, Necab2, Cntnap2*, and *Fam19a1*. Non-peptidergic C-fiber nociceptors(NPs) subsets were annotated using *Gfra1, Gfra2, Trpc3, Lpar3, Mrgpra3, Mrgprd, Sst, Il31ra, Nppb, Trpv1, Trpa1, Ret, Scn10a, Scn11a, P2rx3*, and *Plxnc1*. C-fiber peptidergic nociceptors (PEPs) subsets were annotated using *Tac1, Adcyap1, Gal, Kit, Calca, Ntrk1, Trpa1, Scn10a*, and *Scn11a*. Cold thermoreceptor subsets were annotated using *Trpm8, Tac1, Foxp2, Cdh8, Penk*, and *Piezo2*. Finally, C-LTMRs were annotated using *Th, Slc17a8, Fam19a4, P2ry1, Gfra2, Piezo2*, and *Zfp521/ZNF521*.

To avoid having one species dominate the downstream analyses including integration and to account for potential differences in each species’ clustering resolution, we downsampled the number of nuclei to have similar numbers across species at each DRG subtype cluster (e.g., A-LTMRs, PEPs, NPs, C-LTMRs) using the ‘downsample’ argument in the ‘subset()’ function of SeuratV3. These downsampled DGEs were used for cross-species cell-type mapping analyses including MetaNeighbor and Integration.

### MetaNeighbor analysis

MetaNeighbour v1.9.1 (RRID SCR_016727) was used to provide a measure of neuronal subclass and cluster replicability within and across species (Scripts and tutorials are available on GitHub (http://github.com/gillislab/MetaNeighbor)). The mean area under the receiver operator characteristic curve (AUROC) scores from MetaNeighbor were used as a proxy for the quantitative similarity between cell-type pairs. We performed two rounds of MetaNeighbor analysis, first on the combined all species (mouse, guinea pig, cynomolgus monkey, and human)-all cell types dataset and all species-DRG neuron-specific subsets(downsampled). Highly variable genes were identified using the ‘get_variable_genes()’ function, yielding 493 genes for all cell types dataset and 390 genes for the DRG neuron subset. These were used as input for the ‘MetaNeighbourUS()’ function, which was run using the fast_version. AUROCs are plotted in heat maps in Fig. 3c and Supplementary Fig. 3c. In all species-all cell types MetaNeighbor analysis, the dendrogram of AUROC scores was organized according to major cell types rather than species, suggesting that cell type similarity transcends mammalian species differences (Supplementary Fig. 3c).

### Cross-species dataset integration analysis

To identify homologous cell types across species, we used Seurat’s CCA workflow to perform a separate supervised integration of DRG neurons across species. Downsampled raw expression matrices were reduced to include only those genes with one-to-one orthologues defined in the four species (downloaded from http://www.ensembl.org/biomart/martview)) and placed into Seurat objects with accompanying metadata. To integrate across species, all Seurat objects were merged and normalized using SeuratV3.

### Analysis of core and species-specific transcription profiles

To identify conserved and species-specific transcriptional signatures for each neuron class (i.e., A-LTMRs, PEPs, NPs, C-LTMRs, Cold thermoreceptors), we used expression matrices that were reduced to include only those genes with one-to-one orthologues. Within each species, we performed differential expression(DE) analysis using ‘FindAllMarkers()’ function in SeuratV3 to identify top markers in each neuron subtype/class. While using ‘FindAllMarkers()’ function, we set ‘logfc.threshold’ = 0.5 and ‘min.pct’ = 0.3 for requiring top marker genes to be present in >30% of nuclei and on average, have a log2 fold-difference > 0.5 between two testing groups. We then took the intersection of these DE gene lists to collect those subtype-specific genes that were common across multiple species (Fig. 4c). Figure 4c was created with Customized UpSet Plots codes provided by Chenxin Li [https://github.com/cxli233/customized_upset_plots].

### Divergent genes in the “druggable genome”

To identify genes in the druggable genome group that displays divergent or non-divergent expression patterns between mice and humans, we utilized differentially expressed (DE) gene lists that were used to generate Fig.4c (Supplementary Data 2). From these DE lists, we looked for DE genes that displayed mismatched DRG neuron subtypes between mice and humans (categorized as “divergent”). Then we tabulated how many of these “divergent” genes belonged to different GO categories (GO:0004714, GO:0000981, GO:0005102, GO:0048018, GO:0005216, GO:0004930) in the druggable genome group (Fig. 5f). We also tabulated the number of divergently expressed genes between mouse and human samples from Fig. 5f by DRG classes and subtypes (Fig. 5g).

### Transcriptome correlation analysis

To investigate transcriptome similarity from mouse to human, we performed correlation analysis on gene expression data. Here, for each species we determined the average expression of individual genes by subtype by first summing and averaging all transcript counts then log-transformed the data with the ‘log2()’ function in R. Then, between each pair of species and for each subtype, we calculated the correlation of average gene expression across all genes using the ‘cor()’ function with the ‘method’ argument set to ‘spearman. To further assess the relationships of preclinical models to humans, we plotted the transcriptome correlation by subtype for each species in relation to humans (transcriptome correlation metrics between each species and human are indicated by the dots in Fig. 4d).

### Gene ontology analysis

Gene ontology enrichment analysis was performed using the ‘enrichGO()’ function from the clusterProfiler R package in which *p*-values are calculated based on the hypergeometric distribution and corrected for testing of multiple biological processes GO terms using the Benjamini–Hochberg procedure^44^. GO terms were accessed using the AnnotationHub R package.

### Fluorescent in situ hybridization (RNAScope)

DRGs from mice and guinea pigs were obtained as described above. The nerves and connective tissues were trimmed, and DRGs were placed in OCT molds and frozen rapidly in a mixture of dry ice and Ethanol. Frozen DRGs from cynomolgus monkeys and humans were obtained as described above and mounted in OCT. Frozen human spinal cord samples were obtained from Anabios, assessed for tissue integrity, and prepared for sectioning similar to human DRGs. 5–10 micron DRG and/or spinal cord sections from each species were cut using a cryostat and placed on slides for downstream fluorescence in situ hybridization.

RNAScope Multiplex Fluorescent Kit v2 (Advanced Cell Diagnostics) was used per the manufacturer’s recommendations for fresh-frozen samples with the following alterations. During pretreatment, sections were treated with hydrogen peroxide for 10 min and Protease IV for 20 min prior to the addition of relevant probes. Opal690, 570, and 520 dyes (Akoya Biosciences) were used for fluorescence and after the final HRP block step, samples were stained with DAPI solution for 1 min followed by mounting with ProLong Gold Antifade Mounting Solution (Thermo Fisher Scientific, Cat#P36961). Probes used for RNAscope (Advanced Cell Diagnostics) include: For mouse: Rgs5 (Cat #430181), Th (Cat #317621), Slc17a8 (Cat #431261), Tafa4 (Cat #813621), Ntrk2 (Cat #423611), P2rx3 (Cat #521611), and Tubb3 (Cat #423391). For guinea pig: *TAFA4* (custom, Cat #1128881), NTRK2 (custom, Cat #1128891), P2RX3 (Cat #1201481), mouse Tubb3 used for the pan-neuronal marker. For cynomolgus monkey: TAFA4 (custom, Cat#1128871), NTRK2 (Cat#424151), used human SNAP25 probe for the pan-neuronal marker (Cat #518851). For humans: TAFA (custom, Cat#1037841), NTRK2 (Cat#402621), CDH9 (Cat #403021), SCN5A (Cat #430281), and TUBB3 (Cat#318901). A 3-plex Negative Control Probe (Cat #320871) was used for negative control images for each probe combination per species.

### Fluorescence microscopy and image analysis

Epifluorescence images were taken using a Zeiss Axio Imager.M2 upright microscope equipped with an Apotome.2 structured illumination modules. Images were acquired using a Zeiss Colibri 7 LED, DAPI/AF488/AF555/Cy5 filter sets, a Plan-Apochromat ×20/0.8 objective lens, and a Hamamatsu ORCA-Flash 4.0 Digital CMOS camera. Five z-stack images were collected at 2 μm intervals and maximum intensity projections were created with Zeiss Zen software. For comparison of mouse C-LTMR1 and C-LTMR2 populations, all images were acquired using the same LED intensity and exposure time settings. For cross-species *NTRK2/TAFA4(FAM19A4)/[TUBB3* or *SNAP25]* and C-LTMR proportion (mouse, guinea pig, cynomolgus monkey: *P2RX3/TAF4[TUBB3* or *SNAP25]*; human: *SCN5A/CDH9/TUBB3*) imaging, the image acquisition settings were the same for the set of images within a given species. Human spinal cord image acquisition settings were kept the same as for human DRG when using the *CDH9/SCN5A* probe combination.

All image analysis was performed in ImageJ/FIJI using custom macros. For comparison of mouse C-LTMR1 and C-LTMR2 subtypes, manual regions-of-interest (ROIs) were drawn using the *Slc17a8* signal to mark the entire population of putative C-LTMRs in each section. The optical density of the *Slc17a8*, *Rgs5*, and *Th* channels were measured within each ROI as well as the average optical density of putative single transcripts, identified as distinct, round puncta with clearly decaying intensity on all sides, for each channel per section. Following background correction, the number of puncta per ROI was calculated by dividing the optical density of a given ROI by the average optical density of a single puncta. All measurements were normalized to the cross-sectional area of a given ROI and are reported as the number of puncta per μm^2^. We manually determined a threshold for *Th* high and low cells (dotted line in Fig. 2f) and separated the cells into putative C-LTMR1 and C-LTMR2 populations, respectively, based on the snRNAseq analysis.

For the cross-species comparison of *NTRK2*/TAFA4(*FAM19A4)* expression, neuronal ROIs were segmented automatically with the Cellpose algorithm^45^ for mouse, guinea pig, and cynomolgus macaque using the *TUBB3/SNAP25* images. For humans, neuronal ROIs were drawn manually using the *TUBB3* and DAPI signals due to interference of pervasive autofluorescence/lipofuscin that appeared as a diffuse signal across the AF488/AF55/Cy5 channels, even in the negative control probe images. For mouse, guinea pig, and cynomolgus macaque, a similar analysis pipeline as described above was applied where the optical density for each channel within the neuronal ROIs was measured along with the average optical density of putative single transcripts for each probe. The number of puncta was normalized to the cross-sectional area per neuronal ROI and a threshold for *NTRK2*-positive and *FAM19A4*-positive cells was manually determined for each species. For human images, thresholds for *NTRK2*-positive and *FAM19A4*-positive cells were manually defined based on exceeding any autofluorescence and background signal observed in negative control images. Each individual neuronal ROI was manually defined as *NTRK2*-positive, *FAM19A4*-positive, double-positive, or double-negative.

For the cross-species validation of C-LTMR abundance, probe combinations of *P2RX3/TAFA4/[TUBB3* or *SNAP25]* for mouse, guinea pig, and cynomolgus monkey, or SCN5A/CDH9/*TUBB3* for human DRG were used based on marker gene analysis from the snRNAseq datasets. A similar image analysis workflow as described above was performed including automated neuron segmentation for mouse, guinea pig, and cynomolgus monkey or manual neuron segmentation for human DRG. For mouse, guinea pig, and cynomolgus monkey, the same analysis pipeline was applied to determine the puncta of each probe per cross-sectional area, and a threshold for *P2RX3*-positive and *TAFA4*-positive cells was manually determined for each species. For human images, thresholds for *SCN5A*-positive and *CDH9*-positive cells were defined as above and each neuronal ROI was manually defined as *P2XR3*-positive, *CDH9*-positive, double-positive, or double-negative. Data in Supplementary Fig. 6 was collected from lumbar DRGs and averaged across sections per biological sample (i.e. animal): 2–3 sections per mouse from 2 male and 2 female mice, 2 sections per guinea pig from 2 female guinea pigs, 2 sections per cynomolgus macaque from 3 female macaques, and 2 sections per human from 2 male and 2 female humans. DRG neurons in mice, guinea pigs, and cynomolgus monkeys were considered to be C-LTMRs if they were *TAFA4*-positive and *P2RX3*-negative based on marker gene expression in these species. DRG neurons in humans were considered to be C-LTMRs if they were *CDH9*-positive and *SCN5A*-negative. We did not observe any neurons that met these criteria across 1965 human DRG neurons although rare double-positive neurons were observed.

To validate the human *CDH9* RNAscope probe given the low number of *CDH9-*positive DRG neurons observed, we performed RNAscope with *CDH9/SCN5A* probes on frozen human spinal cord sections based on a single-nuclei RNAseq dataset for human spinal cord^46^ demonstrating expression of these transcripts in neuronal populations. Multiple *CDH9*-positive and some *SCN5A*-positive cells were observed in the human spinal gray matter. Three biological replicates (individual human organ donors; 2 females and 1 male) were used and representative images are displayed in Supplementary Fig. 6b.

All representative images are pseudo-colored with the brightness and contrast adjusted to improve visualization. C-LTMR or neuronal ROIs are overlayed in cyan. For human DRG images in Fig. 5b and Supplementary Fig. 6a, autofluorescence/lipofuscin signals are denoted by white asterisks.

### Statistics and reproducibility

For RNAscope experiments comparing C-LTMR populations in the mouse, three DRG sections per mouse were imaged per vertebral level (cervical, thoracic, lumbar) and the values were averaged across technical replicates; data in Fig. 2f represent each independent biological replicate. DRGs from 2 male and 2 female mice were analyzed and we did not detect any difference between the sexes therefore the data was combined. Data in Fig. 2g is combined for all *Slc17a8*-defined ROIs across all replicates for each vertebral level. The images in Fig. 2e are representative of the data across technical and biological replicates. *Rgs5* area-normalized puncta per cell differences were analyzed using a mixed-effects model with Bonferonni’s multiple comparisons post-test between C-LTMR1 and C-LMTR2.

For RNAscope experiments comparing *TAFA4/NTRK2* expression, data in Fig. 5c was collected from lumbar DRGs and averaged across sections per biological sample (i.e. animal): 3 sections per mouse from 2 male and 2 female mice, 3 sections per guinea pig from 2 female guinea pigs, 2 sections per cynomolgus macaque from 3 female macaques, and 2–3 sections per human from 2 male and 2 female humans. Histograms in Fig. 5d were generated using cross-sectional area data from all *NTRK2*-positive, *TAFA4*-positive, and *TUBB3*-positive ROIs for each respective species. Relative frequency histograms were generated and Gaussian curves were fit using GraphPad Prism v9. Gray dotted lines in each histogram represent the 33rd and 67th percentiles of the entire *TUBB3* distribution for each species as an approximation of small, medium, and large neurons in each species. The images in Fig. 5b are representative of the data across technical and biological replicates per species.

For RNAscope experiments comparing C-LTMR abundance across species, data in Supplementary Fig. 6 was collected from lumbar DRGs and averaged across sections per biological sample (i.e. animal): 2–3 sections per mouse from 2 male and 2 female mice, 2 sections per guinea pig from 2 female guinea pigs, 2 sections per cynomolgus macaque from 3 female macaques, and 2 sections per human from 2 male and 2 female humans. The images in Supplementary Fig. 6a are representative of the data across technical and biological replicates. For the human spinal cord, 1 section per human across three biological replicates (individual human organ donors) was used and images displayed in Supplementary Fig. 6b are representative of data across biological replicates.

### Reporting summary

Further information on research design is available in the Nature Portfolio Reporting Summary linked to this article.

## Supplementary information

Supplementary Information

Description of Additional Supplementary Files

Supplementary Data 1

Supplementary Data 2

Reporting Summary

## Data Availability

The single-nuclei RNA-seq datasets generated in this study have been deposited in the Gene Expression Omnibus (GEO) repository under accession number GSE201654. The Processed data for browsing gene expression in the cross-species data can be assessed under the website: XSpeciesDRGAtlas [http://research-pub.gene.com/XSpeciesDRGAtlas/].The reference genomes used in this study are publicly available: GRCm38 [https://www.ncbi.nlm.nih.gov/assembly/GCF_000001635.20/]; Cavpor3.0 [https://uswest.ensembl.org/Cavia_porcellus/Info/Index]; macFas5 [https://nov2020.archive.ensembl.org/Macaca_fascicularis/Info/Index]; hg19 [https://www.ncbi.nlm.nih.gov/assembly/GCF_000001405.13/].The RNAScope data generated in this study are provided in the Source Data file. Source data are provided with this paper.

## Source data

Source Data

## Code availability

All analyses are based on previously published code and software (see Reporting summary).
