# Supplementary material for: Cross-species transcriptomic atlas of dorsal root ganglia reveals species-specific programs for sensory function
Source: Nat Commun. 2023 Jan 23;14:366. doi: 10.1038/s41467-023-36014-0 (PMC9870891; doi:10.1038/s41467-023-36014-0)
Supplement: Supplementary file 1 — Supplementary Information [file 41467_2023_36014_MOESM1_ESM.pdf]

Supplementary Information

Cross-species transcriptomic atlas of dorsal root ganglia reveals species-specific programs for sensory function

Jung & Dourado et al.

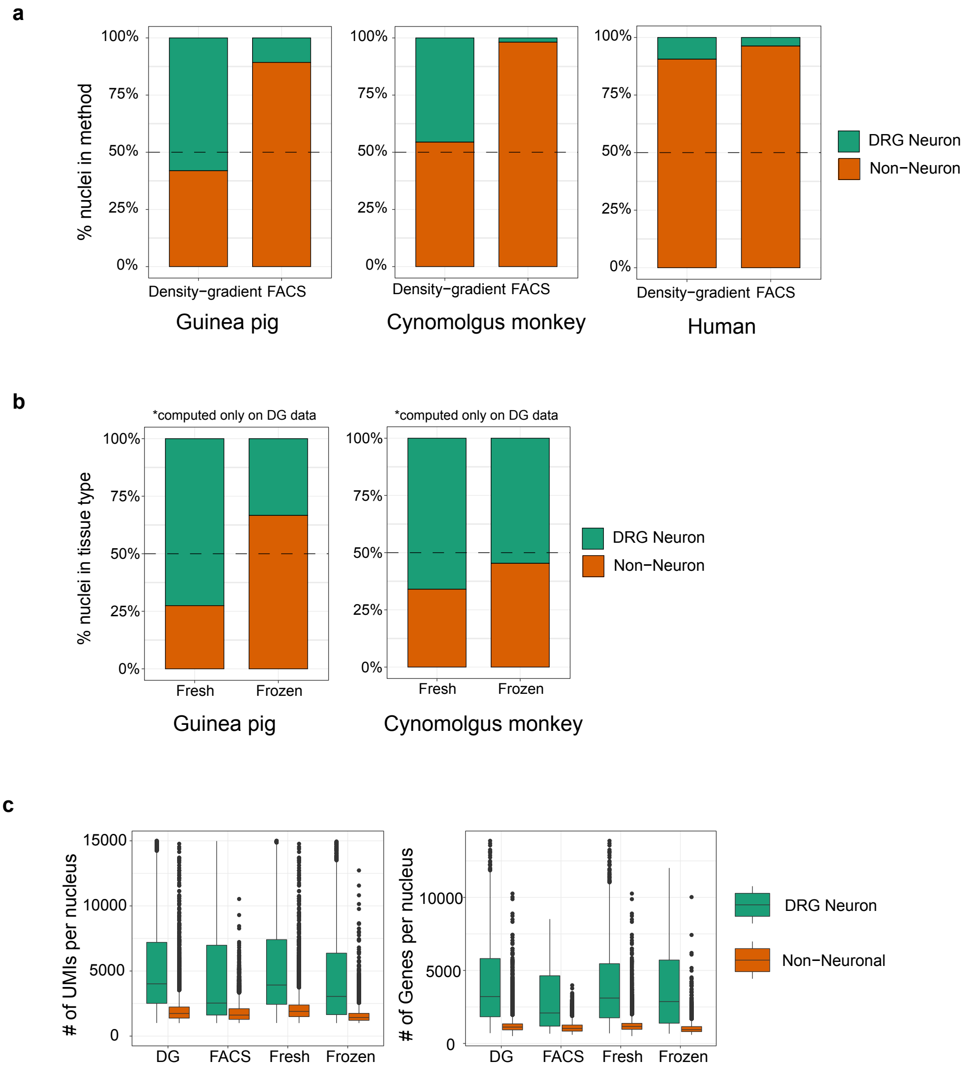

Supplementary Fig. 1 Comparison of nuclei isolation method and tissue types for dorsal root ganglia single-nucleus RNA-seq. a,b Relative cell-type composition for the nuclei isolation methods or tissue types in human, cynomolgus monkey, and guinea pig DRG data. **c** Number of UMIs and genes detected per nucleus for different nuclei isolation methods and tissue types in mouse DRG data. n=37,384 nuclei examined over 10 independent experiments. The lower and upper hinges of the boxes correspond to the 25th to 75th percentile with the line in the middle depicting the median. The whiskers are set at the minimum and maximum values of the dataset. Density-gradient is abbreviated as DG.

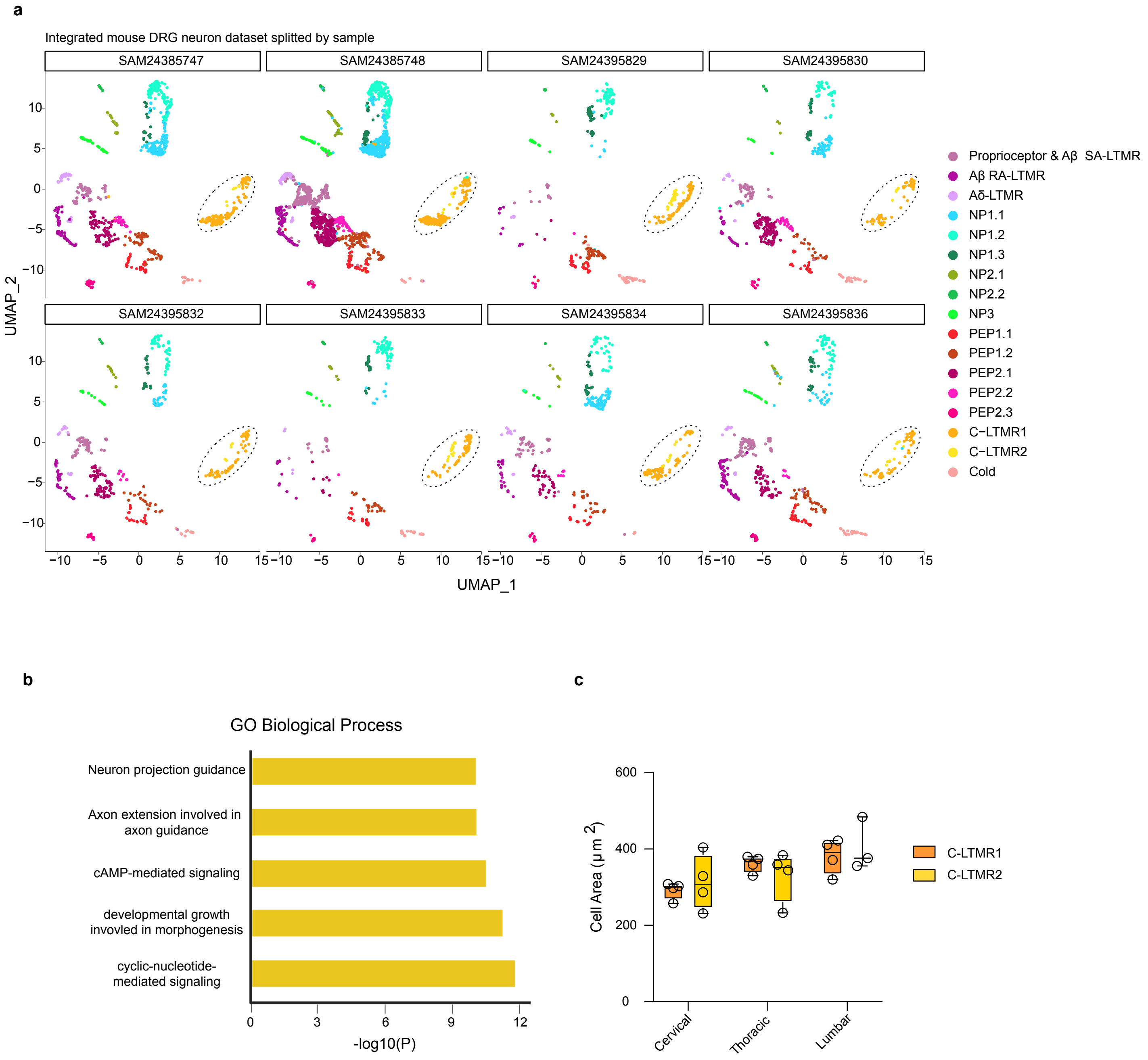

Supplementary Fig. 2 single-nucleus RNA-seq analysis of mouse C-LTMRs a Integrated mouse DRG snRNA-seq data faceted by samples. Dotted circles indicated C-LTMR clusters in each sample. NP denotes non-peptidergic; PEP denotes peptidergic. **b** Gene ontology analysis of genes enriched in the C-LTMR2 subtype. **c** Cell size distribution of C-LTMR subtypes by mouse DRG levels. N = 4 mice. The lower and upper hinges of the boxes correspond to the 25th to 75th percentile with the line in the middle depicting the median. The whiskers are set at the minimum and maximum values of the dataset. Source data are provided as a Source Data file.

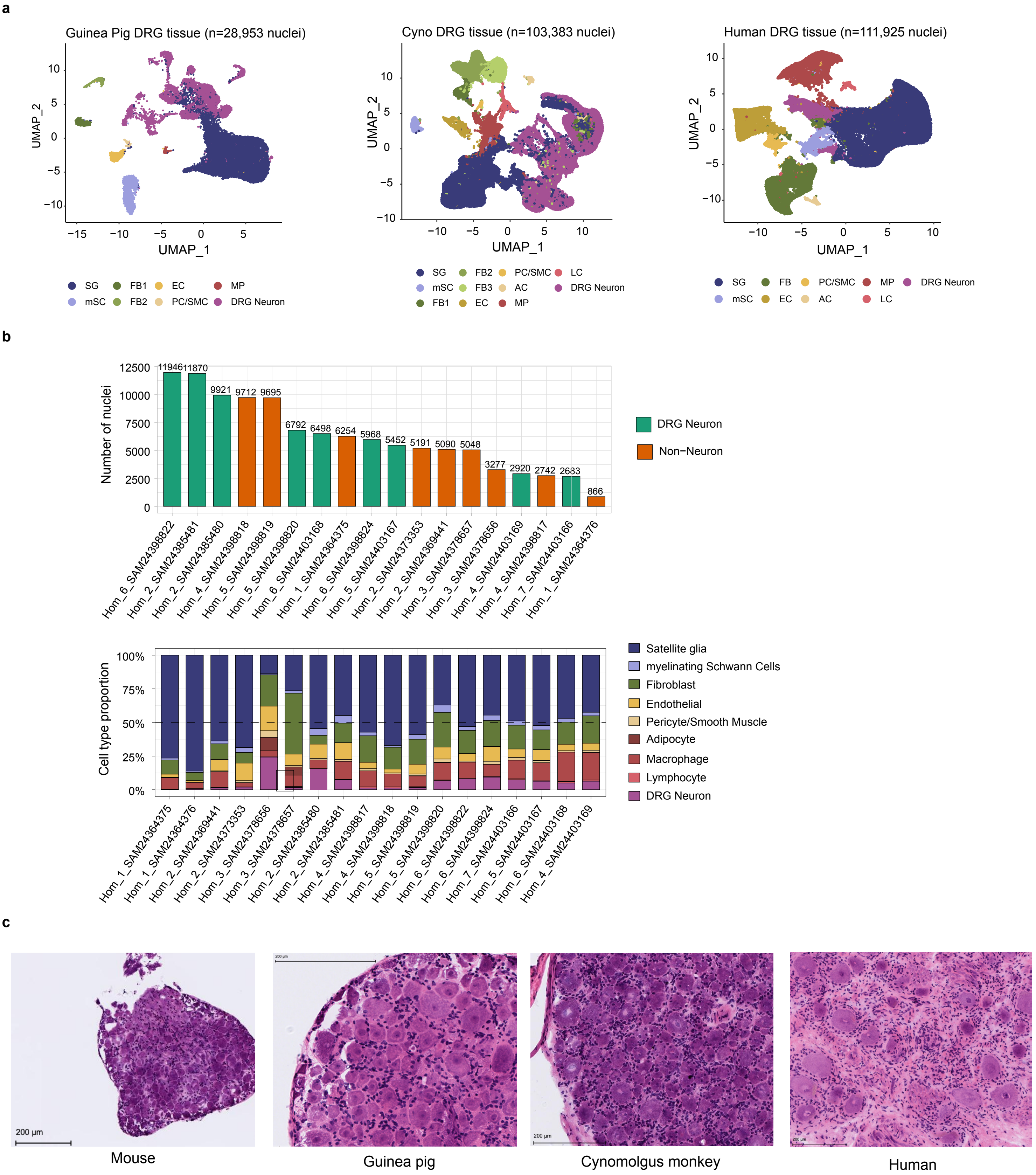
a
Guinea Pig DRG tissue (n=28,953 nuclei)
Cyno DRG tissue (n=103,383 nuclei)
Human DRG tissue (n=111,925 nuclei)
UMAP_1
UMAP_2
SG mSC FB1 FB2 EC PC/SMC MP DRG Neuron
SG mSC FB1 FB2 FB3 PC/SMC AC EC MP LC DRG Neuron
SG mSC FB EC PC/SMC AC MP LC DRG Neuron
b
Number of nuclei
DRG Neuron
Non−Neuron
11946 11870 9921 9712 9695 6792 6498 6254 5968 5452 5191 5090 5048 3277 2920 2742 2683 866
Hom_6_SAM24398822
Hom_2_SAM24385481
Hom_2_SAM24385480
Hom_4_SAM24398818
Hom_5_SAM24398819
Hom_5_SAM24398820
Hom_6_SAM24403168
Hom_1_SAM24364375
Hom_6_SAM24398824
Hom_5_SAM24403167
Hom_2_SAM24373353
Hom_2_SAM24369441
Hom_3_SAM24378657
Hom_3_SAM24378656
Hom_4_SAM24403169
Hom_4_SAM24398817
Hom_7_SAM24403166
Hom_1_SAM24364376
Cell type proportion
Satellite glia
myelinating Schwann Cells
Fibroblast
Endothelial
Pericyte/Smooth Muscle
Adipocyte
Macrophage
Lymphocyte
DRG Neuron
c
Mouse
Guinea pig
Cynomolgus monkey
Human
200 μm

Supplementary Fig. 3 Single-nuclei RNA-seq atlas of dorsal root ganglia from guinea pig, cynomolgus monkey, and human. a UMAP of guinea pig, cynomolgus monkey, and human DRG nuclei colored by cell types and annotated post hoc. We profiled 28,953 nuclei from guinea pig DRGs, 103,383 nuclei from cynomolgus monkey DRGs, and 111,925 nuclei from human DRGs. SC denotes satellite glia; mSC denotes myelinating Scwhann Cells; FB denotes fibroblasts; EC denotes endothelial cells; SMC/PC denotes smooth muscle cells and pericytes; AC denotes adipocytes; MP denotes macrophages; LC denotes lymphocytes. **b** Number of nuclei and cell type proportions recovered from human donors. **c** H&E images of DRG tissues from mouse, guinea pig, cynomolgus monkey and human with scale bars. The H&E experiment was repeated with 3 different samples for each species with similar results.

a

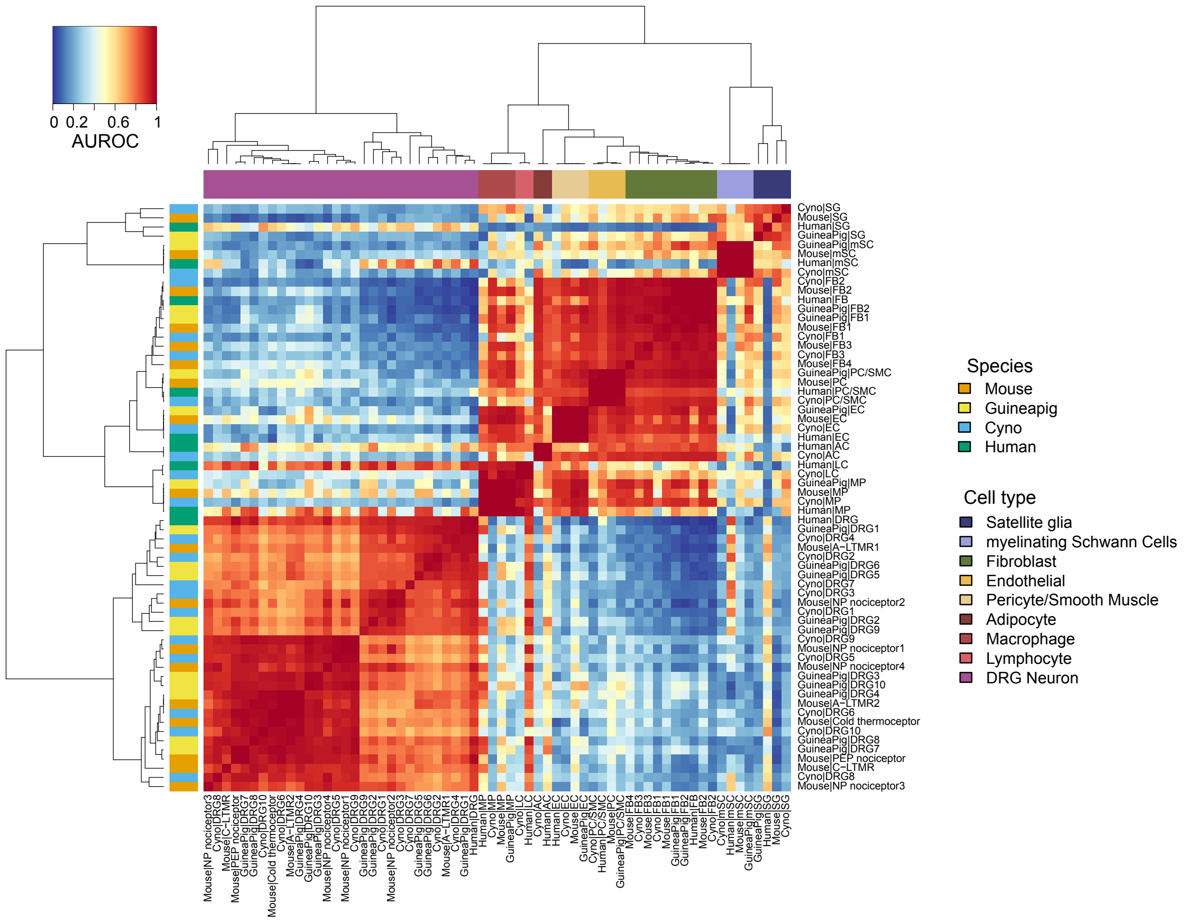

b

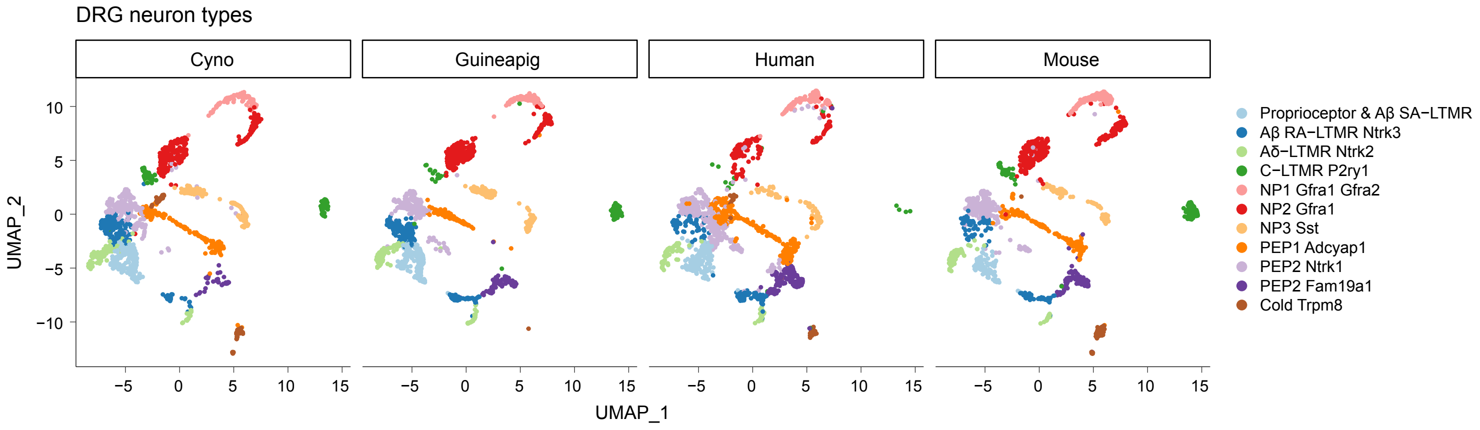

Supplementary Fig. 4 Integrative cross-species single-nuclei RNA-seq analysis of dorsal root ganglia from guinea pig, cynomolgus monkey, and human. a Cross-species dendrogram and heatmap of MetaNeighbor mean AUROC(area under the receiver operator characteristic curve) scores (y-axis in plot) color bar indicates cell types in DRGs (top) and species (left). **b** UMAP of Seurat-integrated DRG neurons across mouse, guinea pig, cynomolgus monkey, and human, faceted by species and colored by DRG neuron subtypes. NP denotes non-peptidergic; PEP denotes peptidergic.

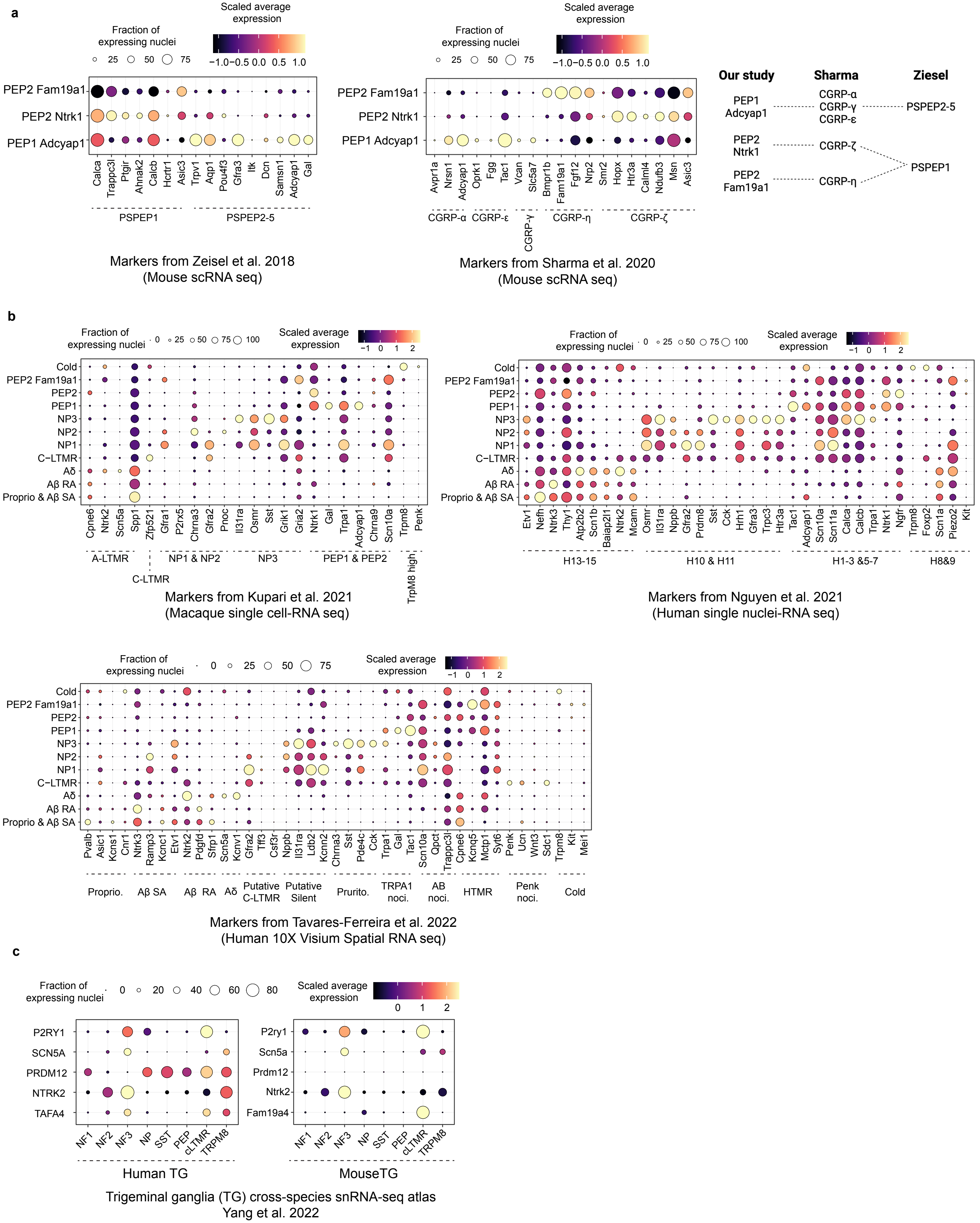
a
Fraction of expressing nuclei
25 50 75
Scaled average expression
−1.0 −0.5 0.0 0.5 1.0
PEP2 Fam19a1
PEP2 Ntrk1
PEP1 Adcyap1
Calca Trappc3l Ptgir Ahnak2 Calcb Hcrtr1 Asic3 Trpv1 Aqp1 Pou4f3 Gfra3 Itk Dcn Samsn1 Adcyap1 Gal
PSPEP1
PSPEP2-5
Markers from Zeisel et al. 2018
(Mouse scRNA seq)
Avpr1a Nrsn1 Adcyap1 Oprk1 Fgg Tac1 Vcan Slc5a7 Bmpr1b Fam19a1 Fgf12 Nrp2 Smr2 Hopx Htr3a Calml4 Ndufb3 Msn Asic3
CGRP-α CGRP-ε CGRP-γ CGRP-η CGRP-ζ
Markers from Sharma et al. 2020
(Mouse scRNA seq)
Our study
Sharma
Ziesel
PEP1 Adcyap1
CGRP-α CGRP-γ CGRP-ε
PSPEP2-5
PEP2 Ntrk1
CGRP-ζ
PSPEP1
PEP2 Fam19a1
CGRP-η
b
Fraction of expressing nuclei
0 25 50 75 100
Scaled average expression
−1 0 1 2
Cold
PEP2 Fam19a1
PEP2
PEP1
NP3
NP2
NP1
C−LTMR
Aδ
Aβ RA
Proprio & Aβ SA
Cpne6 Ntrk2 Scn5a Spp1 Zfp521 Gfra1 P2rx5 Chrna3 Gfra2 Pnoc Il31ra Osmr Sst Grik1 Gria2 Ntrk1 Gal Trpa1 Adcyap1 Chrna9 Scn10a Trpm8 Penk
A-LTMR
C-LTMR
NP1 & NP2
NP3
PEP1 & PEP2
TrpM8 high
Markers from Kupari et al. 2021
(Macaque single cell-RNA seq)
Etv1 Nefh Ntrk3 Thy1 Atp2b2 Scn1b Baiap2l1 Ntrk2 Mcam Osmr Il31ra Nppb Gfra2 Prdm8 Sst Cck Hrh1 Gfra3 Trpc3 Htr3a Tac1 Adcyap1 Scn10a Scn11a Calca Calcb Trpa1 Ntrk1 Ngfr Trpm8 Foxp2 Scn1a Piezo2 Kit
H13-15
H10 & H11
H1-3 &5-7
H8&9
Markers from Nguyen et al. 2021
(Human single nuclei-RNA seq)
Fraction of expressing nuclei
0 25 50 75
Scaled average expression
−1 0 1 2
Pvalb Asic1 Kcns1 Cnr1 Ntrk3 Ramp3 Kcnc1 Etv1 Ntrk2 Pdgfd Sfrp1 Scn5a Kcnv1 Gfra2 Tff3 Csf3r Nppb Il31ra Ldb2 Kcnn2 Chrna3 Sst Pde4c Cck Trpa1 Gal Tac1 Scn10a Qpct Trappc3l Cpne6 Kcnq5 Mctp1 Syt6 Penk Ucn Wnt3 Sdc1 Trpm8 Kit Mei1
Proprio.
Aβ SA
Aβ RA
Aδ
Putative C-LTMR
Putative Silent
Prurito.
TRPA1 noci.
AB noci.
HTMR
Penk noci.
Cold
Markers from Tavares-Ferreira et al. 2022
(Human 10X Visium Spatial RNA seq)
c
Fraction of expressing nuclei
0 20 40 60 80
Scaled average expression
0 1 2
P2RY1
SCN5A
PRDM12
NTRK2
TAFA4
NF1 NF2 NF3 NP SST PEP cLTMR TRPM8
Human TG
P2ry1
Scn5a
Prdm12
Ntrk2
Fam19a4
MouseTG
Trigeminal ganglia (TG) cross-species snRNA-seq atlas
Yang et al. 2022

Supplementary Fig. 5 Expression of markers from external sensory neuron expression data. **a-c** Fraction of nuclei (dot size) in each neuron subtype expressing markers from external sensory neuron studies (columns) and their scaled average expression level in expressing nuclei (dot color). NP denotes non-peptidergic; PEP denotes peptidergic. **a** Expression of peptidergic marker genes defined by external mouse DRG studies in our mouse peptidergic cell populations data. We identified that: our PEP2 Ntrk1 cluster corresponds to CGRP-ζ (Sharma) and PSPEP1 (Zeisel) clusters; our PEP2 Fam19a1 cluster corresponds to CGRP-η (Sharma) and PSPEP1 (Zeisel) clusters; and our PEP1 Adcyap1 cluster corresponds to CGRP-α, ε and γ (Sharma) and PSPEP2-5 (Zeisel).
b Expression of marker genes from recently published primate analyses in our primate data.
c Expression of TAFA4, Aδ and C-LTMR specific markers (as identified in this study) shown in recently published human and mouse trigeminal ganglion single-nuclei RNA-seq atlas.

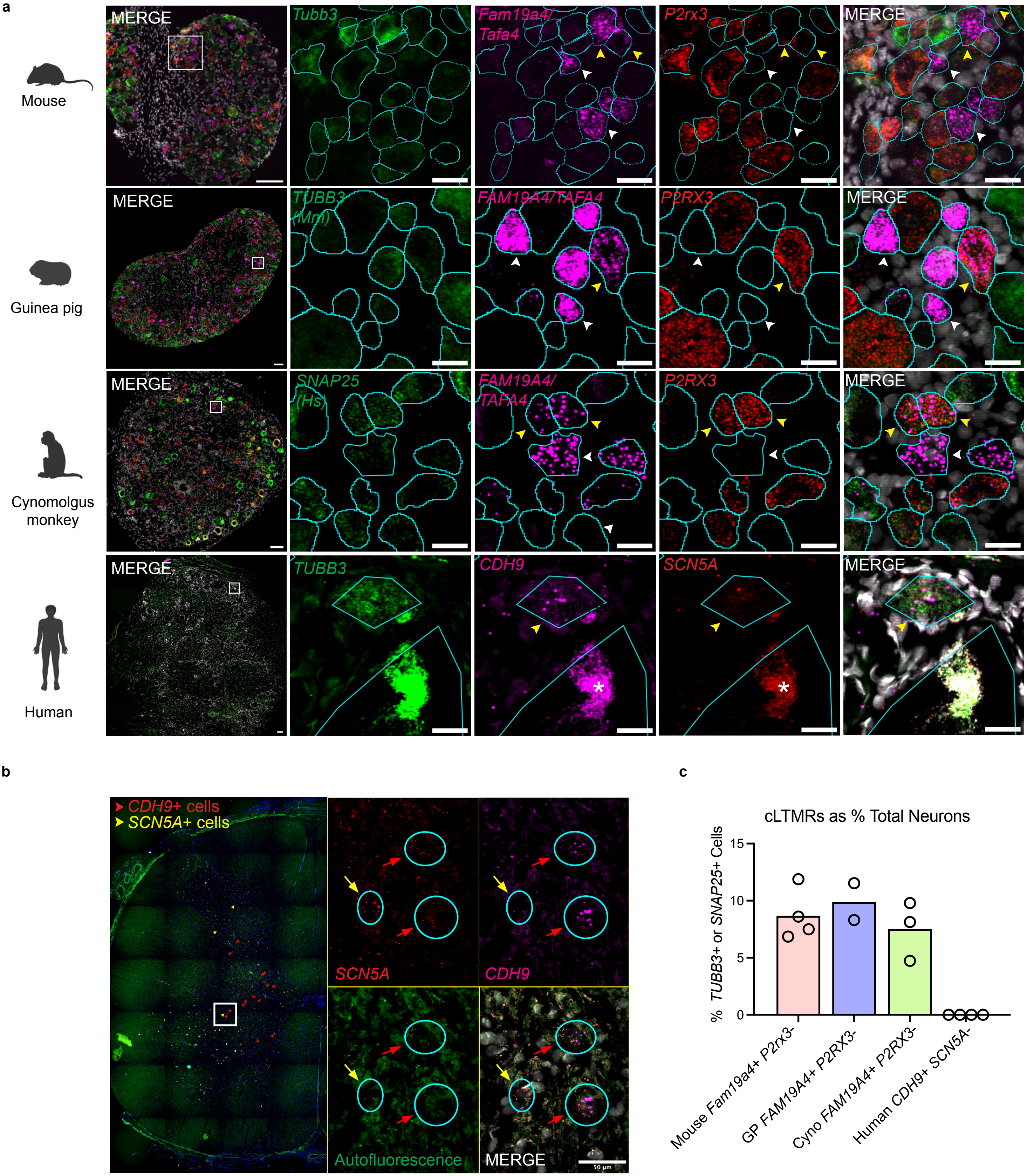
a
Mouse
Guinea pig
Cynomolgus monkey
Human
MERGE
Tubb3
Fam19a4/Tafa4
P2rx3
TUBB3 (Mm)
FAM19A4/TAFA4
P2RX3
SNAP25 (Hs)
CDH9
SCN5A
b
CDH9+ cells
SCN5A+ cells
SCN5A
CDH9
Autofluorescence
MERGE
50 μm
c
cLTMRs as % Total Neurons
% TUBB3+ or SNAP25+ Cells
15
10
5
0
Mouse Fam19a4+ P2rx3-
GP FAM19A4+ P2RX3-
Cyno FAM19A4+ P2RX3-
Human CDH9+ SCN5A-

Supplementary Fig. 6 RNAScope validation of C-LTMRs in mouse, guinea pig, cynomolgus monkey, and human DRGs. a RNAScope validation of *TUBB3* (mouse, guinea pig, and human) or *SNAP25* (cynomolgous monkey), *FAM19A4*, and *P2RX3* (mouse, guinea pig, cynomolgous monkey) or *CDH9* and *SCN5A* (human) in DRGs. The scale bar in the whole DRG image indicates 100µm. Neurons outlined in turquoise. The scale bar in the zoomed images indicate 25µm. Yellow arrowheads indicate *FAM19A4*+ and *P2RX3*+ or *SCN5A* cells. White arrowheads indicate *FAM19A4*+ and *P2RX3*- cells. Asterisks in the human images indicate lipofuscin aggregates. **b** Positive control of *CDH9* probe in human spinal cord tissue. Yellow arrows indicate SCN5A+ cells and red arrows indicate *CDH9*+ cells. Autofluorescence reflects signal from the AF488 channel.The scale bar indicates 50µm. **c** Percentage of *FAM19A4+/P2RX3*- or *CDH9+/SCN5A*-cells (putative C-LTMRs) in mouse, guinea pig, cynomolgus monkey, human. No *CDH9*+ cells were observed in human DRGs**.** Source data are provided as a Source Data file. GP denotes guinea pig and Cyno denotes cynomolgus monkey. Species icons in **a** were created with BioRender.com

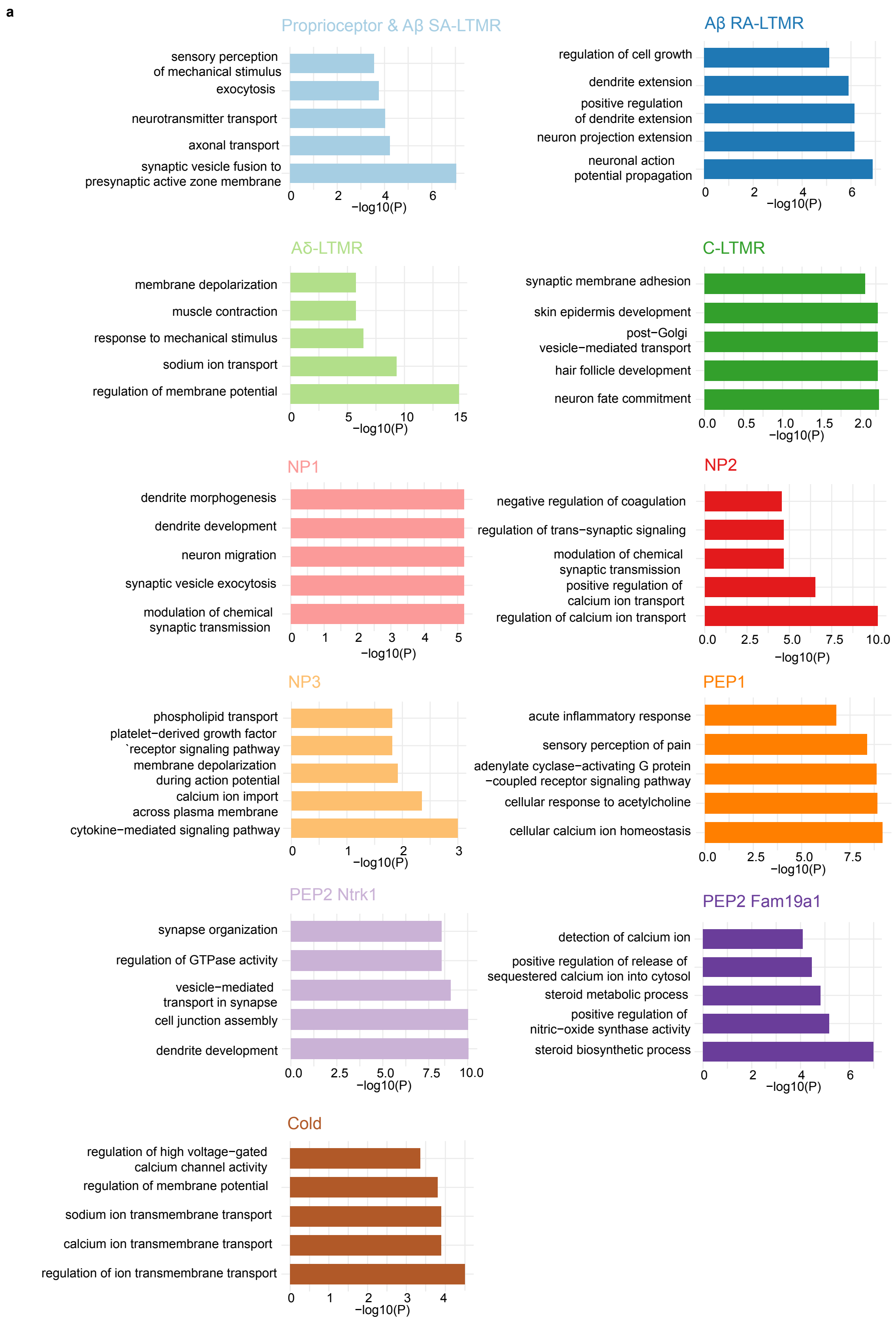

Supplementary Fig. 7 Analysis of conserved DRG neuron subtype-specific transcriptional programs. a Gene ontology (Biological processes) analysis of subtype-specific conserved genes. p-values are calculated based on the hypergeometric distribution which corresponds to one-sided Fisher's exact test and corrected for multiple comparisons testing using the Benjamini-Hochberg. NP denotes non-peptidergic; PEP denotes peptidergic.

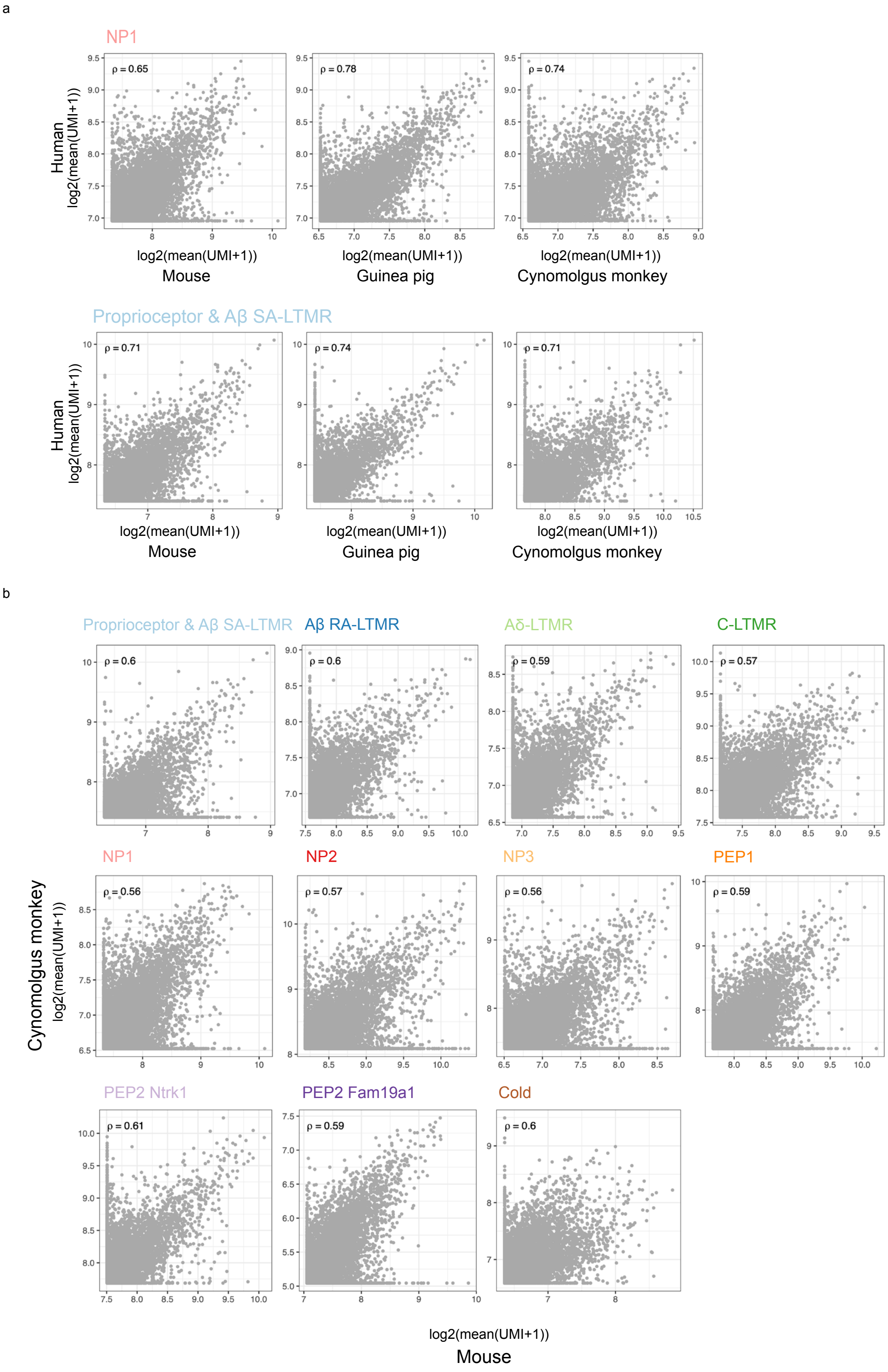

Supplementary Fig. 8 Analysis of conserved DRG neuron subtype-specific transcriptional programs. a Scatter plots of transcriptome correlation (Spearman) between human and other species for NP1 and Proprioceptor & Aβ SA-LTMRs. NP denotes non-peptidergic **b** Scatter plots of transcriptome correlation (Spearman) between cynomolgus monkey and mouse for each DRG subtype. NP denotes non-peptidergic; PEP denotes peptidergic.

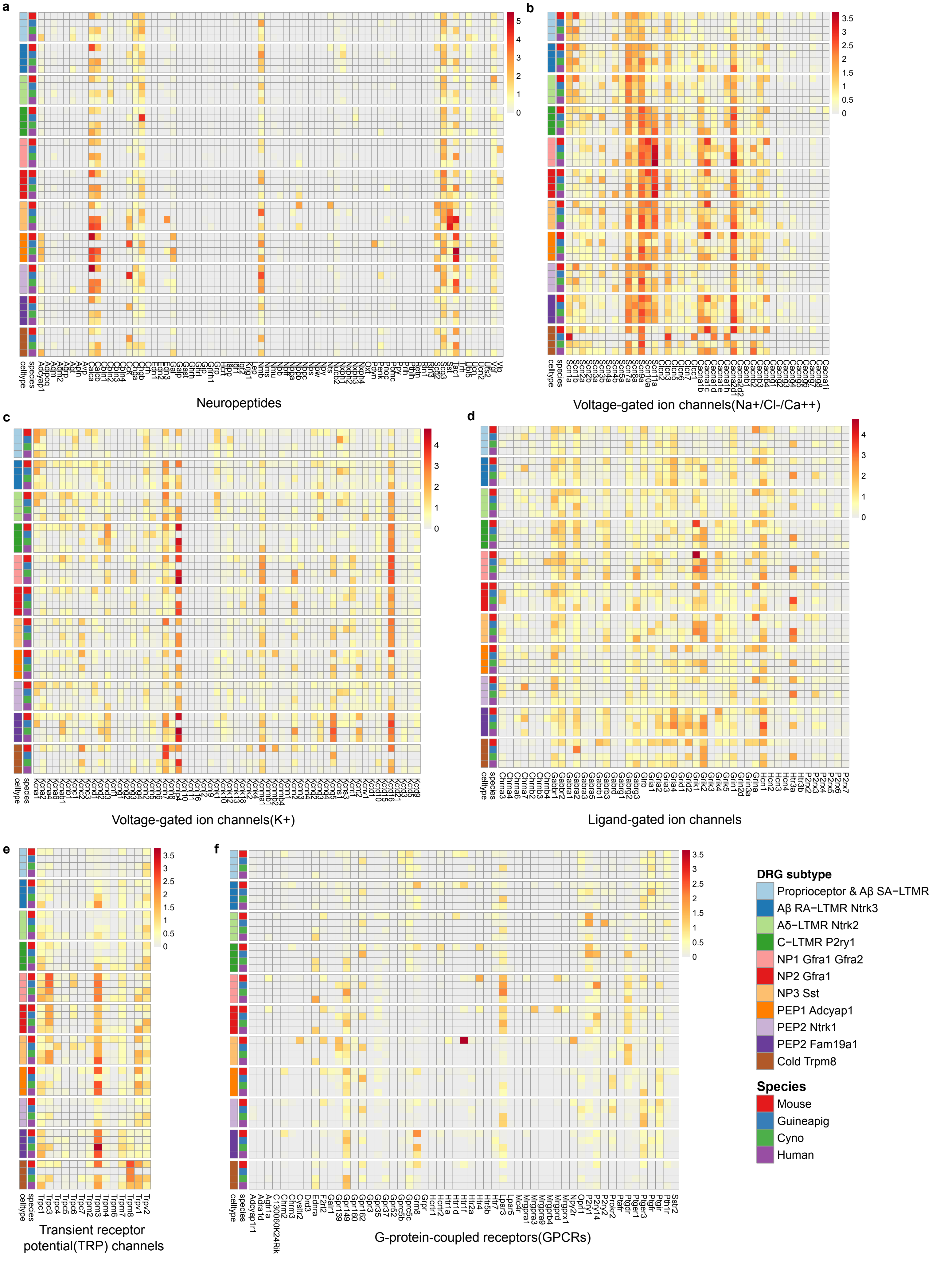

a
Neuropeptides
b
Voltage-gated ion channels(Na+/Cl-/Ca++)
c
Voltage-gated ion channels(K+)
d
Ligand-gated ion channels
e
Transient receptor potential(TRP) channels
f
G-protein-coupled receptors(GPCRs)
celltype
species
DRG subtype
Proprioceptor & Aβ SA−LTMR
Aβ RA−LTMR Ntrk3
Aδ−LTMR Ntrk2
C−LTMR P2ry1
NP1 Gfra1 Gfra2
NP2 Gfra1
NP3 Sst
PEP1 Adcyap1
PEP2 Ntrk1
PEP2 Fam19a1
Cold Trpm8
Species
Mouse
Guineapig
Cyno
Human

Supplementary Fig. 9 Expression of molecular determinants of sensory function

a-f Expression heatmap of neuropeptides, voltage-gated ion channels, transient receptor potential channels, ligand-gated ion channels and G-protein coupled receptors in each neuron subtype in mouse, guinea pig, cynomolgus monkey, and human data. NP denotes non-peptidergic; PEP denotes peptidergic.

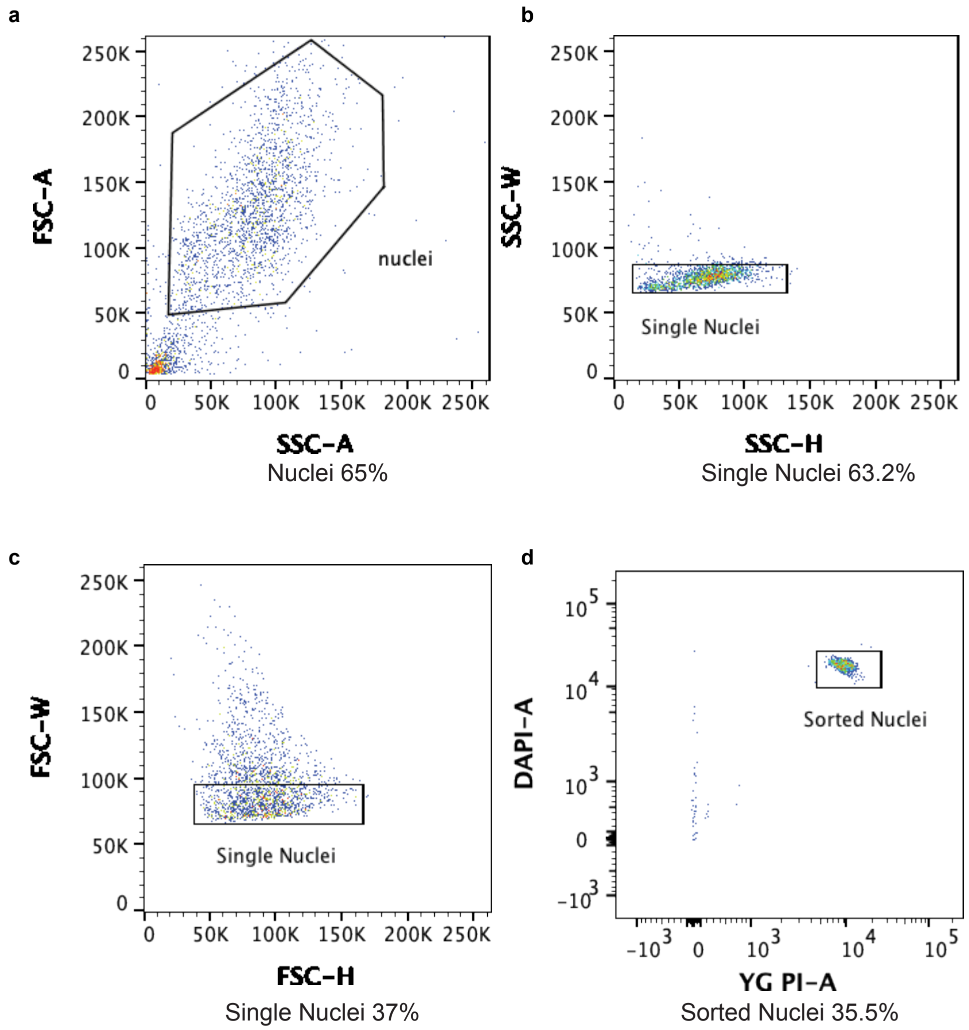

Supplementary Fig. 10 Flow cytometry gating strategy for fsorted single nuclei.
Single nuclei were sorted on a BD FACSAria Fusion with BD FACSDiva Software v8.0.3. The instrument was set to sort in "Purity" mode with a system pressure of 70psi on a 70 micron nozzle at 32kHz frequency. The fraction of gated nuclei is listed below each plot as a percentage of total. **a** Primary gate to select nuclei. **b** Secondary gate selecting singlets based on side scatter. **c** Tertiary gate selecting singlets based on forward scatter. **d** Sorted single nuclei selected for sequencing based on DAPI and propidium iodide (PI) labeling.
