# Supplementary material for: Cross-species transcriptomic atlas of dorsal root ganglia reveals species-specific programs for sensory function
Source: Nat Commun. 2023 Jan 23;14:366. doi: 10.1038/s41467-023-36014-0 (PMC9870891; doi:10.1038/s41467-023-36014-0)
Supplement: Supplementary file 2 — Description of Additional Supplementary Files [file 41467_2023_36014_MOESM2_ESM.pdf]

Description of Additional Supplementary Files

File Name: Supplementary Data 1
Description: Summary of samples profiled in this study. Description of each DRG sample profiled in this study, including species, age, sex, tissue archival method, nuclei isolation method, and DRG collection location.

File Name: Supplementary Data 2
Description: DRG neuron subtype-specific DE genes for mouse, guinea pig, cynomolgus monkey and human. Differential expression statistics summarizing the marker genes for DRG neuron subtypes for each species (mouse, guinea pig, cynomolgus monkey and human).
