# Supplementary material for: Cross-species transcriptomic atlas of dorsal root ganglia reveals species-specific programs for sensory function
Source: Nat Commun. 2023 Jan 23;14:366. doi: 10.1038/s41467-023-36014-0 (PMC9870891; doi:10.1038/s41467-023-36014-0)
Supplement: Supplementary file 5 — Reporting Summary [file 41467_2023_36014_MOESM5_ESM.docx]

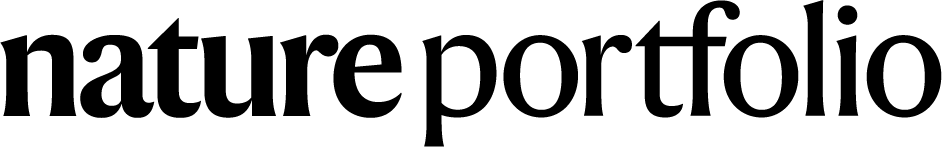
Corresponding author(s):

Joshua S. Kaminker, David H. Hackos, Lorena Riol-Blanco

1

nature portfolio | reporting summary

*March 2021*

Last updated by author(s): Jan 6, 2023

Reporting Summary

Nature Portfolio wishes to improve the reproducibility of the work that we publish. This form provides structure for consistency and transparency in reporting. For further information on Nature Portfolio policies, see our Editorial Policies and the Editorial Policy Checklist.

Please do not complete any field with "not applicable" or n/a. Refer to the help text for what text to use if an item is not relevant to your study. For final submission: please carefully check your responses for accuracy; you will not be able to make changes later.

## Statistics

For all statistical analyses, confirm that the following items are present in the figure legend, table legend, main text, or Methods section. n/a Confirmed


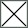

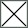

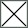


The exact sample size (*n*) for each experimental group/condition, given as a discrete number and unit of measurement

A statement on whether measurements were taken from distinct samples or whether the same sample was measured repeatedly The statistical test(s) used AND whether they are one- or two-sided

*Only common tests should be described solely by name; describe more complex techniques in the Methods section.*

A description of all covariates tested

A description of any assumptions or corrections, such as tests of normality and adjustment for multiple comparisons

A full description of the statistical parameters including central tendency (e.g. means) or other basic estimates (e.g. regression coefficient) AND variation (e.g. standard deviation) or associated estimates of uncertainty (e.g. confidence intervals)

For null hypothesis testing, the test statistic (e.g. *F*, *t*, *r*) with confidence intervals, effect sizes, degrees of freedom and *P* value noted

*Give P values as exact values whenever suitable.*

For Bayesian analysis, information on the choice of priors and Markov chain Monte Carlo settings

For hierarchical and complex designs, identification of the appropriate level for tests and full reporting of outcomes Estimates of effect sizes (e.g. Cohen's *d*, Pearson's *r*), indicating how they were calculated

*Our web collection on statistics for biologists contains articles on many of the points above.*

## Software and code

Policy information about availability of computer code Data collection

CellRanger5.0, Zeiss Zen Software (v14.0.24.021;2011)

Data analysis

R(v4.1.1), Seurat(v3.2.2), MetaNeighbor(v1.14.0), GSNAP(2013-10-10-v2), CellBender(v.0.2.0) ,edgeR(3.36.0), clusterProfiler(4.2.2), AnnotationHub(3.2.2), CellPose(2.0), GraphPad Prism(v9), FACSDiva(v8.0.3), Customized UpSet Plots(v2)

All analyses were based on previously published code (see below): https://satijalab.org/seurat/articles/integration_introduction.html https://github.com/broadinstitute/CellBender/blob/master/docs/source/introduction/index.rst https://bioconductor.org/packages/release/bioc/html/edgeR.html https://github.com/gillislab/MetaNeighbor https://bioconductor.org/packages/release/bioc/html/clusterProfiler.html https://bioconductor.org/packages/release/bioc/html/AnnotationHub.html https://github.com/MouseLand/cellpose

https://github.com/cxli233/customized_upset_plots

All analyses performed are based on previously published code and software.

For manuscripts utilizing custom algorithms or software that are central to the research but not yet described in published literature, software must be made available to editors and reviewers. We strongly encourage code deposition in a community repository (e.g. GitHub). See the Nature Portfolio guidelines for submitting code & software for further information.

## Data

Policy information about availability of data

All manuscripts must include a data availability statement. This statement should provide the following information, where applicable:

- Accession codes, unique identifiers, or web links for publicly available datasets
- A description of any restrictions on data availability
- For clinical datasets or third party data, please ensure that the statement adheres to our policy

The datasets generated during and/or analysed during the current study are available in the GEO: GSE201654 [(https://www.ncbi.nlm.nih.gov/geo/query/acc.cgi?](http://www.ncbi.nlm.nih.gov/geo/query/acc.cgi) acc=GSE201654). Processed data for browsing gene expression in the cross-species data can be assessed at the following link: [XSpeciesDRGAtlas(http://research-](http://research-/) pub.gene.com/XSpeciesDRGAtlas/)

Reference genomes for different species analyzed in this study are publicly available: [GRCm38(https://www.ncbi.nlm.nih.gov/assembly/GCF_000001635.20/),](http://www.ncbi.nlm.nih.gov/assembly/GCF_000001635.20/)) Cavpor3.0(https://uswest.ensembl.org/Cavia_porcellus/Info/Index), macFas5(https://nov2020.archive.ensembl.org/Macaca_fascicularis/Info/Index), hg19(https:// [www.ncbi.nlm.nih.gov/assembly/GCF_000001405.13/)](http://www.ncbi.nlm.nih.gov/assembly/GCF_000001405.13/))

The RNAScope data generated in this study are provided in the Source Data file.

## Human research participants

Policy information about studies involving human research participants and Sex and Gender in Research.

Reporting on sex and gender

Frozen Human DRG tissues were obtained from Anabios (6 donors total; 2 female donors and 4 male donors) and Donor Network West (1 female donor). We were restricted by the availability of human tissues but we made efforts to include both sexes, if possible. No sex-based analyses were performed in this study.

Population characteristics

We did not make any selection of samples regarding age, genotype information, or other population characteristic features. We requested tissues from healthy donors only. Vendors did not the disclose cause of death for all frozen human tissues samples. Only sex and age associated with all human tissue samples were provided and all other information including Protected Health Information (“PHI”), as defined by HIPAA, have been removed by vendors prior to transfer of tissues to Genentech.

Additional details on individual samples are available in Supplementary Data 1.

Recruitment

We procured the frozen tissues from vendors and had no participation in patient recruitment. Thus, our study design was blinded to any selection-bias in tissues selection.

Ethics oversight

All human tissue samples were supplied by AnaBios Corporation and Donor Network West. Each supplier received IRB approval of research, appropriate informed consent of all subjects contributing biological materials, and all other authorizations, consents or permissions as necessary for the transfer and use of the biological materials for research at Genentech. Patients/human donors were not compensated for their donation of tissues.

Note that full information on the approval of the study protocol must also be provided in the manuscript.

# Field-specific reporting

Please select the one below that is the best fit for your research. If you are not sure, read the appropriate sections before making your selection.

Life sciences

Behavioural & social sciences

Ecological, evolutionary & environmental sciences

For a reference copy of the document with all sections, see nature.com/documents/nr-reporting-summary-flat.pdf

2

nature portfolio | reporting summary

*March 2021*

# Life sciences study design

No sample size calculation was performed to pre-determine sample sizes.

For the snRNA-seq exepriments we used 10 samples from 5 mice, 8 samples from 2 guinea pigs, 16 samples from 3 cynomolgus monkeys, and 18 samples from 7 human donors. These sample sizes were restricted by the availability of animals and tissues. For our snRNA-seq analyses, these numbers of samples are sufficient for obtaining the necessary number of nuclei to perform data analysis and characterization of cell types.

For the RNAScope validation, DRGs from 2 male and 2 female mice were analyzed. As we did not detect any difference between sexes we combined the data for these samples. 3 DRG sections per mouse were imaged per vertebral level (cervical, thoracic, lumbar) and the values were averaged across technical replicates; the data in Fig. 2f represent each independent biological replicate.

All studies must disclose on these points even when the disclosure is negative. Sample size

3

nature portfolio | reporting summary

*March 2021*

Data in Fig. 5c and Supplementary Fig. 6a were collected from lumbar DRGs and averaged across sections per biological sample (i.e. animal): 3

sections per mouse from 2 male and 2 female mice, 3 sections per guinea pig from 2 female guinea pigs, 2 sections per cynomolgus macaque from 3 female macaques, and 2-3 sections per human from 2 male and 2 female humans.

Data in Supp. Fig. 6a was collected from lumbar DRGs and averaged across sections per biological sample (i.e. animal): 2-3 sections per mouse from 2 male and 2 female mice, 2 sections per guinea pig from 2 female guinea pigs, 2 sections per cynomolgus macaque from 3 female macaques, and 2 sections per human from 2 male and 2 female humans.

3 biological replicates (individual human organ donors; 2 females and 1 male) were used and representative images are displayed in Supp Fig. 6b.

For RNAscope experiments, sample sizes were estimated based on previous studies for mice and the availability of animals and tissue for guinea pig, cynomolgus macaque, and human. These sample sizes allowed for statistical comparisons to be performed where appropriate and/or validation of results across at least 2 biological replicates.

Data exclusions

For the snRNA-seq experiments we excluded low quality and non-neuornal cells through our quality control pipeline as described in the Methods section. The data were excluded based on thresholds for counts, genes and percentage of mitochondrial reads, as described in Methods. Low quality, injured, and non-neuronal (lacking neuronal markers or expressing high neuronal markers) were omitted to enhance the quality of analysis.

Additional details on data filtering available in Methods.

Replication

All experiments have at least one another replicate. Specific number of replicates varies for snRNA-seq or RNAScope. Detailed information can be accessible in Supplementary Data 1 and Methods. The findings from the computational analysis were successfully validated using RNAScope.

Randomization

Samples were restricted to availability of animals and tissues. There was not a different experimental condition to be tested, so no randomization was needed. Computational analysis did not yield animal or sex specific findings.

Blinding

There was not a different experimental condition to be tested so no blinding was necessary. Our single-nuclei analysis was performed using unbiased computational methods published by others; hence no blinding was necessary.

# Reporting for specific materials, systems and methods

We require information from authors about some types of materials, experimental systems and methods used in many studies. Here, indicate whether each material, system or method listed is relevant to your study. If you are not sure if a list item applies to your research, read the appropriate section before selecting a response.

Materials & experimental systems Methods


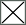

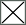

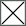

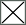

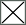


n/a Involved in the study Antibodies Eukaryotic cell lines

Palaeontology and archaeology Animals and other organisms Clinical data

Dual use research of concern


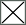

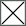


n/a Involved in the study

ChIP-seq

Flow cytometry

MRI-based neuroimaging

## Animals and other research organisms

Policy information about studies involving animals; ARRIVE guidelines recommended for reporting animal research, and Sex and Gender in Research

Laboratory animals

5 C57BL/6J mice (Mus musculus), female and males, 8-17 weeks old; 2 female Hartley guinea pigs (Cavia porcellus), 5.5-6 months old; 3 female crab- eating macaques (Macaca fascicularis), 8-9 years old.

The laboratory mice were kept on a 12-h light/dark cycle at the controlled room temperature of 20–22 °C with humidity of 40–50% for the duration of the experiments.

Wild animals

No wild animals were used in this study.

Reporting on sex

Sex was not considered in this study design as tissue sample selection was based on the availability of animals. We did not perform sex-based analyses.

Field-collected samples

No field-collected samples were used in this study.

Ethics oversight

Care and handling procedures of animals were reviewed and approved by the Genentech Institutional Animal Care and Use

4

nature portfolio | reporting summary

*March 2021*

Ethics oversight

Committee (IACUC) and animal experiments were conducted in full compliance with IACUC policies and NIH guidelines.

Note that full information on the approval of the study protocol must also be provided in the manuscript.

## Flow Cytometry

### Plots

Confirm that:

The axis labels state the marker and fluorochrome used (e.g. CD4-FITC).

The axis scales are clearly visible. Include numbers along axes only for bottom left plot of group (a 'group' is an analysis of identical markers). All plots are contour plots with outliers or pseudocolor plots.

A numerical value for number of cells or percentage (with statistics) is provided.

### Methodology

Sample preparation

Dounce homogenization was used to dissociate all DRG tissues. Before douncing, 1 mL HBSS (ThermoFisher, Catalog #14025092) containing 3% BSA Fraction VI and RNAse inhibitor in nuclei suspension buffer (NSB) was added to the 2 mL Dounce Tissue Grinder (Kimble Chase, Catalog #885300-0002). The DRGs were homogenized with an A (“loose”) pestle using 5 to 10 strokes. The homogenate was then filtered through a 70-micron filter and spun down. The pellet was resuspended in the NSB. The frozen DRGs were not allowed to thaw before placing in the lysis buffer and douncing. The homogenate was layered over an Optiprep density (Sigma, Catalog #D-1556; 35%, 16%, 8% for rodents and 40%, 20% and 10% for primates) gradient and centrifuged at 2500 g for 20 min. Nuclei at the 16/35 interface were aspirated and mixed with an equal volume of NSB. Prior to sorting, nuclei were re-pelleted, by centrifugation, labeled with propidium iodide (Life Technologies, Catalog #P1304MP) and DAPI (Life Technologies Catalog #62248)

Instrument

FACS Aria Fusion Flow Cytometer; BD Biosciences

Software

Data were collected and analyzed using BD FACSDiva Software v8.0.3

Cell population abundance

90-100% of post-sorting nuclei were DAPI and PI positive nuclei. For each sample, we sorted ~50,000 nuclei for performing droplet generation for snRNA-seq.

Gating strategy

All nuclei were stained with DAPI and PI. First, debris were excluded using FSc/SSc. Then nuclei were selected based on double labeling with DAPI and PI. The instrument was set to sort in "Purity" mode with a system pressure of 70psi on a 70 micron nozzle at 32kHz frequency.

Tick this box to confirm that a figure exemplifying the gating strategy is provided in the Supplementary Information.


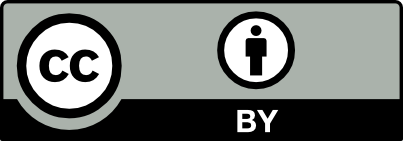
This checklist template is licensed under a Creative Commons Attribution 4.0 International License, which permits use, sharing, adaptation, distribution and reproduction in any medium or format, as long as you give appropriate credit to the original author(s) and the source, provide a link to the Creative Commons license, and indicate if changes were made. The images or other third party material in this article are included in the article's Creative Commons license, unless indicated otherwise in a credit line to the material. If material is not included in the article's Creative Commons license and your intended use is not permitted by statutory regulation or exceeds the permitted use, you will need to obtain permission directly from the copyright holder. To view a copy of this license, visit <http://creativecommons.org/licenses/by/4.0/>
